# CRISPR/Cas9-Mediated *SlNPR1* mutagenesis reduces tomato plant drought tolerance

**DOI:** 10.1186/s12870-018-1627-4

**Published:** 2019-01-22

**Authors:** Rui Li, Chunxue Liu, Ruirui Zhao, Liu Wang, Lin Chen, Wenqing Yu, Shujuan Zhang, Jiping Sheng, Lin Shen

**Affiliations:** 1 College of Food Science and Nutritional Engineering, China Agricultural University, Beijing, 100083 China; 2 School of Agricultural Economics and Rural Development, Renmin University of China, Beijing, 100872 China

**Keywords:** CRISPR/Cas9, *SlNPR1*, Drought, ROS, Stomatal closure, Tomato plant

## Abstract

**Background:**

*NPR1*, nonexpressor of pathogenesis-related gene 1, is a master regulator involved in plant defense response to pathogens, and its regulatory mechanism in the defense pathway has been relatively clear. However, information about the function of *NPR1* in plant response to abiotic stress is still limited. Tomato is the fourth most economically crop worldwide and also one of the best-characterized model plants employed in genetic studies. Because of the lack of a stable tomato *NPR1* (*SlNPR1*) mutant, little is known about the function of *SlNPR1* in tomato response to biotic and abiotic stresses.

**Results:**

Here we isolated *SlNPR1* from tomato ‘Ailsa Craig’ and generated *slnpr1* mutants using the CRISPR/Cas9 system. Analysis of the *cis*-acting elements indicated that *SlNPR1* might be involved in tomato plant response to drought stress. Expression pattern analysis showed that *SlNPR1* was expressed in all plant tissues, and it was strongly induced by drought stress. Thus, we investigated the function of *SlNPR1* in tomato-plant drought tolerance. Results showed that *slnpr1* mutants exhibited reduced drought tolerance with increased stomatal aperture, higher electrolytic leakage, malondialdehyde (MDA) and hydrogen peroxide (H_2_O_2_) levels, and lower activity levels of antioxidant enzymes, compared to wild type (WT) plants. The reduced drought tolerance of *slnpr1* mutants was further reflected by the down-regulated expression of drought related key genes, including *SlGST*, *SlDHN*, and *SlDREB*.

**Conclusions:**

Collectively, the data suggest that *SlNPR1* is involved in regulating tomato plant drought response. These results aid in further understanding the molecular basis underlying *SlNPR1* mediation of tomato drought sensitivity.

**Electronic supplementary material:**

The online version of this article (10.1186/s12870-018-1627-4) contains supplementary material, which is available to authorized users.

## Background

Drought is one of the harshest environmental factors limiting plant growth, development, and survival [1]. Due to global warming, drought has become an issue requiring an urgent solution in agricultural production [2]. Tomato (*Solanum lycopersicum*) is an important vegetable crop cultivated around the world, but its most economical cultivars are highly sensitive to drought [3, 4]. Thus, a more in-depth exploration of tomato plant drought tolerance regulatory mechanisms is the most attractive and feasible option to alleviate the loss in drought-affected environments.

There have been identified a range of physiological and biochemical pathways, involved in or affected by drought stress [5]. Adverse environmental conditions severely affect plants primarily due to excessive accumulation of reactive oxygen species (ROS) [6]. Antioxidant enzymes including ascorbate peroxidase (APX), superoxide dismutase (SOD), peroxidase (POD), and catalase (CAT), play critical roles in coping with continuous ROS production [7, 8]. Electrolyte leakage and malondialdehyde (MDA) accumulation can indicate cell membrane damage from drought stress [9].

Nonexpressor of pathogenesis-related gene 1 (*NPR1*, also known as *NIM1*), a special receptor of salicylic acid (SA), is considered as an integral part in systemic acquired resistance (SAR) [10]. NPR1 is a conserved protein with Broad-Complex, Tramtrack, and Bric-a-brac/poxvirus and Zinc finger (BTB/POZ) domain; and Ankyrin-repeat domain, both of which are essential for protein-protein interactions and for enabling NPR1 to function as a co-activator [11]. Phylogenetic analysis revealed that there are three functionally distinct clades of the NPR1-like protein family [12]. Members of the clade including AtNPR1 and AtNPR2 often positively participate in SAR regulation [12, 13]. However, members of the clade including AtNPR3 and AtNPR4 are always associated with negative SAR regulation, yet are required in mounting SAR [14]. In addition, AtBOP1 and AtBOP2 belonging to another clade are associated with the development of lateral organs [15].

Previous reports have shown that *Arabidopsis thaliana NPR1* (*AtNPR1*) positively regulates plant response to biotic stress [16, 17]. Before infection, NPR1 protein is in an oxidized oligomeric form in the cytoplasm [17]. Once the pathogens infect, SA accumulation leads to a change in intracellular redox potential, which enables NPR1 to translocate into the nucleus and interact with TGA-bZIP transcription factors to activate multiple pathogenesis-related (PR) genes [18, 19]. Overexpression of *AtNPR1* or its orthologs enhances disease resistance in transgenic *A. thaliana* [13], carrots [20], citrus [21], apple [22], and grapevine [23] plants. However, information about NPR1’s implication in plant response to abiotic stress is still limited [24]. Recent report in *A. thaliana* has showed that AtNPR1 is involved in the cold acclimation through interacting with HSFA1 factors [24]. NPR1-dependent SA signaling pathway is crucial for enhancing tolerance to salt and oxidative stresses in *A. thaliana* [25]. Heterologous expression of *AtNPR1* in tobacco plant can enhance the tolerance to oxidative stress [26]. Moreover, a suppressed *MdNPR1* transcription is shown in the leaves of drought-treated apple trees [27]. In contrast, overexpression of *AtNPR1* in rice is shown to confer hypersensitivity to salt and drought stresses [28]. These apparently contradictory results question the role of *NPR1* gene in plant drought-tolerance mediation.

Tomato is a very popular crop because of its great nutritive and commercial values, and it is also often used to study gene function [29]. Thus, to further improve our understanding of the function of *NPR1* in plants, it is necessary to characterize *SlNPR1*’s functions in tomato plant drought tolerance. In this study, we isolated *SlNPR1* from tomato ‘Ailsa Craig’, investigated its expression profile in all plant tissues and under drought stress. The clustered regularly interspaced short palindromic repeats (CRISPR)/ CRISPR-associated protein-9 nuclease (Cas9) technology has been used in various fields of research and commercial development in basic science, medicine, and agriculture because of its high efficiency, low cost, and design flexibility [30]. We used bioinformatics analysis to predict the function of *SlNPR1*, and then generated the *slnpr1* mutants using the CRISPR/ Cas9 system. Furthermore, to discover a possible regulatory mechanism mediated by *SlNPR1*, we compared the drought tolerance of *slnpr1* mutants (L16, L21, and L62) and wild type (WT) plants at physiological and molecular levels by analyzing stomatal closure, membrane damage, antioxidant-enzyme activities, and drought-related gene expression. These results provide information on underlying *SlNPR1* mediation drought regulatory mechanism in tomato plants.

## Results

### Bioinformatics analysis

**Fig. 1:**
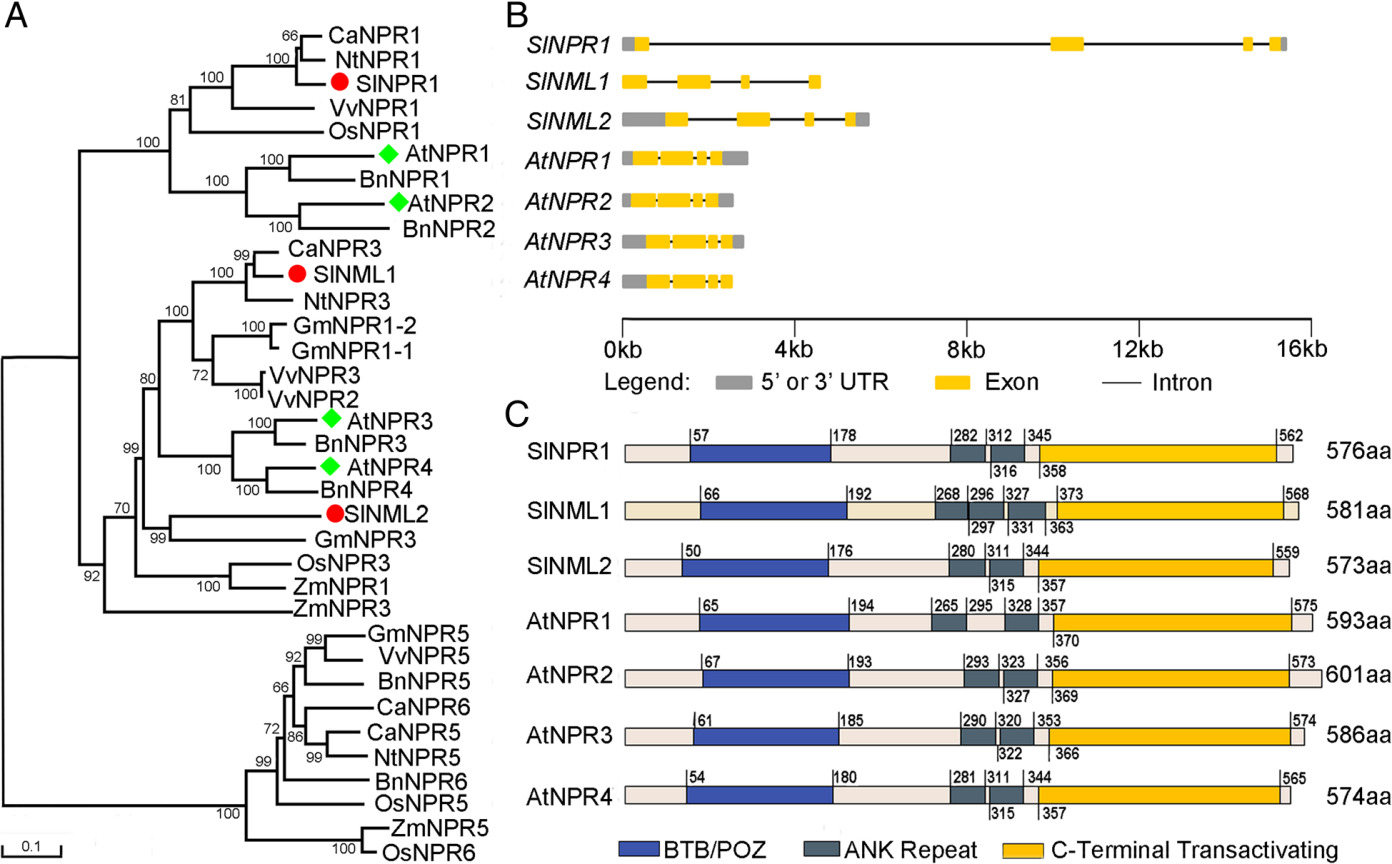
Phylogenetic, gene structure, and domain analyses of *SlNPR1*. (**a**) Phylogenetic tree of 35 plant NPR1 homologous proteins identified from nine plant species (MEGA 5.0; Neighbour-Joining (NJ) method; bootstrap of 1000). (**b**) Exon/intron structure and (**c**) domain organization of NPR proteins identified from tomato and *Arabidopsis thaliana*. The domains and motifs are drawn to scale. Among them, the unmarked pink areas don’t code any known domain.

*SlNPR1* was cloned from *Solanum lycopersicum* ‘Ailsa Craig’ and sequenced (Accession no: KX198701). *SlNPR1* consisted of 1731bp, encoding for a putative protein with 576 amino acid residues, a predicted molecular mass of 64.2 kDa, and a calculated pI of 5.70. Three NPR1 homologous proteins from tomato (SlNPR1, SlNML1, and SlNML2), together with 32 NPR1 proteins from other plant species (Additional file 1: Table S1), were subjected to phylogenetic analysis. Results revealed that SlNPR1 was highly similar to NtNPR1 from tobacco (89% identity, 94% similarity) and CaNPR1 from pimento (91% identity, 95% similarity) as well as VvNPR1 from grapevine and OsNPR1 from rice; they all belonged to the clade containing AtNPR1 and AtNPR2 (Fig. 1a). However, SlNML1 and SlNML2 formed a distinct clade with AtNPR3 and AtNPR4, and they were similar to AtNPR3 (58% identity, 73% similarity, and 51% identity, 70% similarity, respectively) (Fig. 1a). Compared to SlNML1 and SlNML2, SlNPR1 showed highest similarity to AtNPR1 (53% identity, 72% similarity).

Exon/intron structure analysis illustrated similarity between *NPR1* homologous genes from tomato and *A. thaliana*. They all contained three introns and four exons. Interestingly, the distance between adjacent exons of tomato *NPR1* was much longer than that in *A. thaliana* (Fig. 1b). Domain composition analysis revealed that NPR1 homologous proteins identified from tomato and *A. thaliana* shared highly conserved domains. They all contained BTB/POZ motif, ANK repeats, and C-terminal trans-activating domain at similar positions (Fig. 1c).

Additionally, SlNPR1’s N-terminal region contains an IκB-like phosphodegron motif (DS×××S), which has been shown to promote NPR1 turnover by phosphorylation of residues Ser11/Ser15 in AtNPR1 [31]. A completely conserved penta-amino acid motif (LENRV) was also found in SlNPR1’s C-terminal region. It serves as a binding site for NIM interacting (NIMIN) 1/2 protein in tobacco [32]. However, AtNPR1’s nuclear localization signal (NLS) sequence motif (KK×R××××××××KK) was not fully conserved in SlNPR1 (Additional file 2: Figure S1).

### *Cis*-acting regulatory elements in *SlNPR1* promoter

Promoter sequence analysis showed that a variety of *cis*-elements, which respond to hormone treatment and biotic stress (Table 1). SA-responsive elements (TCA-element and WBOXATNPR1), MeJA-responsive element (TGACG-motif), pathogen- and GA- responsive element (WRKY71OS), and disease resistance response element (BIHD1OS), were abundant in *SlNPR1*’s promoter region. This was in accordance with previous reports, which showed that NPR1 played a key role in defense response involved in the SA- and/or JA-signaling pathway [33]. Meanwhile, some *cis*-elements, which respond to abiotic stresses, including drought-responsive elements (MYCATRD22 and MYCATERD1), salt and light responsive element (GT-1 motif), ABA-responsive element (ABRE), and heat stress responsive element (HSE), were also found (Table 1). These results suggest that *SlNPR1* might be involved in not only biotic stresses but also abiotic stresses, such as drought stress.

**Table 1:** *Cis*-acting elements present in the *SlNPR1* promoter.

*Cis*-acting elements	Number	Sequence	Characteristic
TC-rich repeats	2	ATTTTCTTCA	Defense and stress responsiveness
MYCATRD22	1	CACATG	MYC recognition site, dehydration responsiveness
MYCATERD1	1	CATGTG	Drought-responsive element
ABRE	2	CACGTG	ABA-responsive element
ARE	1	TGGTTT	Anaerobic induction elements
HSE	2	AAAAAATTTC	Heat stress responsive element
GT-1 motif	3	GAAAAAATGGTGGTTGG	Salt and light responsive element
BIHD1OS	3	TGTCA	Disease resistance responses
WBOXATNPR1	3	TTGAC	Abiotic stress and SA-responsiveness
WRKY71OS	6	TGAC	WRKY binding site, pathogen- and GA-responsiveness
TCA-element	2	GAGAAGAATA	SA-responsive element
TGACG-motif	3	TGACG	MeJA- responsive element
ERE	3	ATTTCAAA	ET-responsive element
TGA-box	1	TGACGTAA	Auxin-responsive element

### Generation of *slnpr1* mutants using the CRISPR/Cas9 gene-editing system

**Fig. 2:**
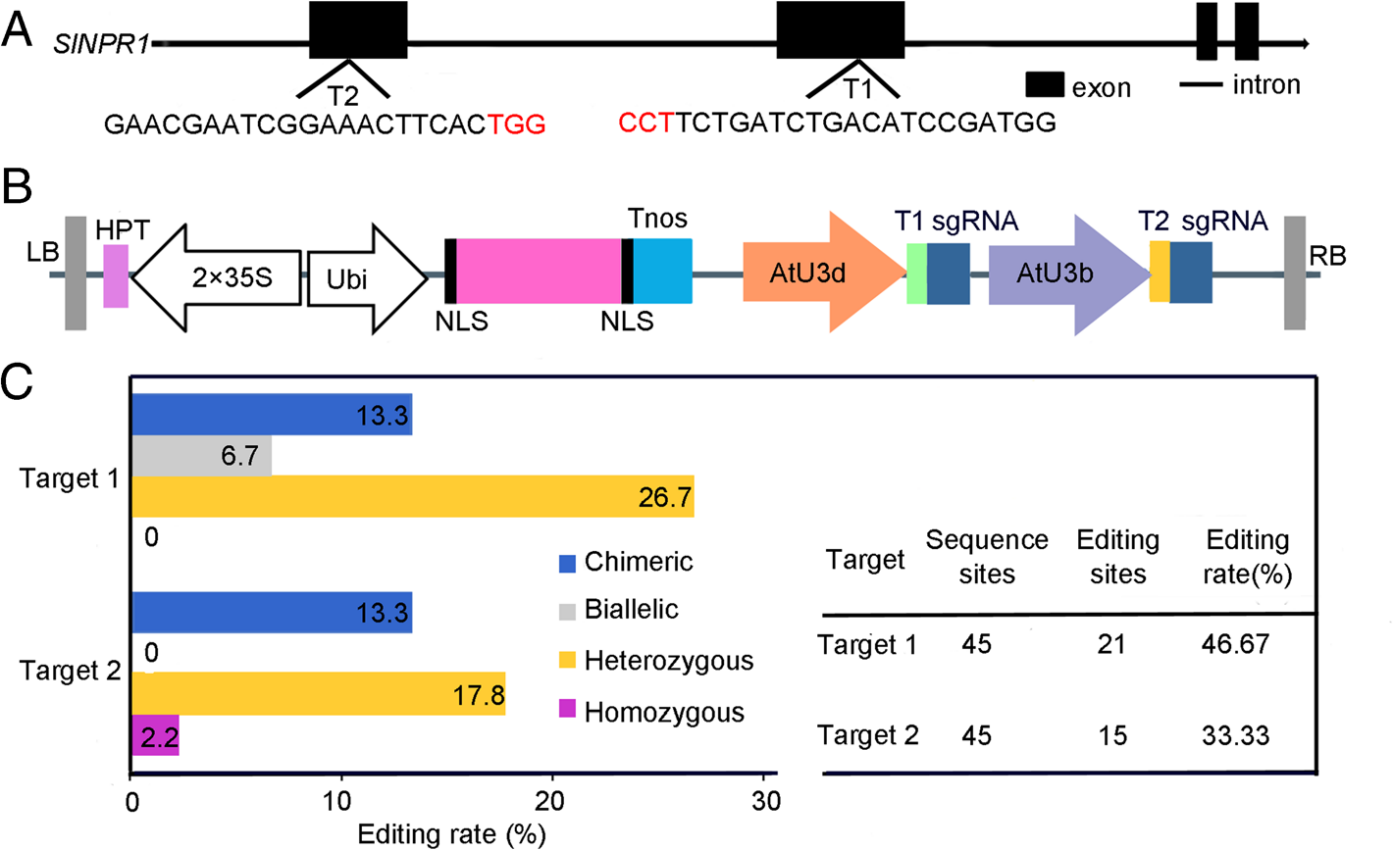
CRISPR/Cas9-mediated genome editing. (**a**) Schematic illustration of the two target sites in *SlNPR1* genomic sequence. Target 1 and target 2 sequences are shown in capital letters and the protospacer adjacent motif (PAM) sequence is marked in red. (**b**) Schematic diagram of pYLCRISPR/Cas9-*SlNPR1* vector. HPT, hygromycin B phosphotransferase; Ubi, maize ubiquitin promoter; NLS, nuclear localization sequence; Tnos, gene terminator; AtU3d, *Arabidopsis thaliana* U3d promoter; AtU3b, *A. thaliana* U3b promoter. (**c**) CRISPR/Cas9-mediated efficient edit and variant genotypes of two target sequences in T0 plants.

To understand the role of *SlNPR1* in a plant’s response to drought stress better, we generated *slnpr1* mutants using the CRISPR/Cas9 gene editing technology. Two target sites Target 1 and Target 2 were designed for *SlNPR1* (Fig. 2a and b), and 45 T0-independent transgenic plants were obtained through *Agrobacterium*-mediated transformation. Furthermore, chimeric, biallelic, heterozygous, and homozygous *slnpr1* mutants were present in the T0 generation. To further verify the editing types of *slnpr1* mutants, these independent transgenic lines were analyzed by sequencing, and the special editing types are listed in Additional file 3: Figure S2. Additionally, editing rates of the two target sequences were 46.67% (Target 1) and 33.33% (Target 2). Among the four editing types, heterozygous mutations were the most common ones (26.7%, Target 1; 17.8%, Target 2) (Fig. 2c and Additional file 3: Figure S2), and the editing sites frequently occurred at about 3 bp upstream from the protospacer adjacent motif (PAM) sequence (Additional file 3: Figure S2) [34]. In addition, majority of the editing types were almost small insertions and deletions at target sites (Additional file 3: Figure S2), which would lead to loss of *SlNPR1* function through frame shift [35].

To investigate whether mutations generated by the CRISPR/Cas9 system could be inherited in the next generation, we randomly selected T1 generation derived from corresponding T0 transgenic lines CR-*NPR1*-16, CR-*NPR1*-21, and CR-*NPR1*-62 (L16, L21, and L62) for editing type analysis (Additional file 3: Figure S2). Among all T1 transgenic plants examined, only one T1 generation transgenic plant derived from L16 was WT. Although two plants derived from L21 failed to edit in Target 2, they were edited in Target 1 (Table 2). Meanwhile, to determine the accuracy of target gene (*SlNPR1*), off-target analysis was performed among T1 generation transgenic lines. The results indicated that no mutations were observed in any potential off-target site in T1 generation plants (Additional file 4: Table S2), which suggested that CRISPR/Cas9-mediated mutagenesis was highly specific for *SlNPR1*. Therefore, the defined T1 generation transgenic plants derived from L16, L21, and L62 were used for the further study.

**Table 2:** Segregation patterns of CRISPRCas9-medicated targeted mutagenesis during the T0 to T1 generation.

Mutant plants	T_0_ generation		Mutation transmission in the T_1_ generation					
	Genotype	Mutation type	No. of plants tested	WT	Bi-allele	Homozygote	Heterozygote	Chimeric
Line 16	(T2) Heterozygote	(wt, i1)	21	1	1 (d3, i1), 1 (d2, i1)	9 (i1)	6 (wt, i1)	3
Line 21	(T1) Heterozygote	(wt, i1)	22	0	2 (i1, d4), 1 (s4, i1), 1 (i1, d5),	6 (i1)	11 (wt, i1)	1
	(T2) Heterozygote	(wt, s3/d4)	22	2	0	7 (d4)	13 (wt, d4),	0
Line 62	(T1) Biallelic	(i1, d4)	20	0	10 (i1, d4), 1 (i1, d8),	3 (d4), 6 (i1)	0	0
	(T2) Heterozygote	(wt, d4)	20	0	2(d3, d4)	5 (d4)	13 (wt, d4)	0

*wt* wild-type sequence without mutations detected at target sequences, *d#* the number of bases deleted from the target sequences, *i#* the number of bases inserted at target sequences, *s#* the number of bases substituted origin target sequences.

### Expression pattern

**Fig. 3:**
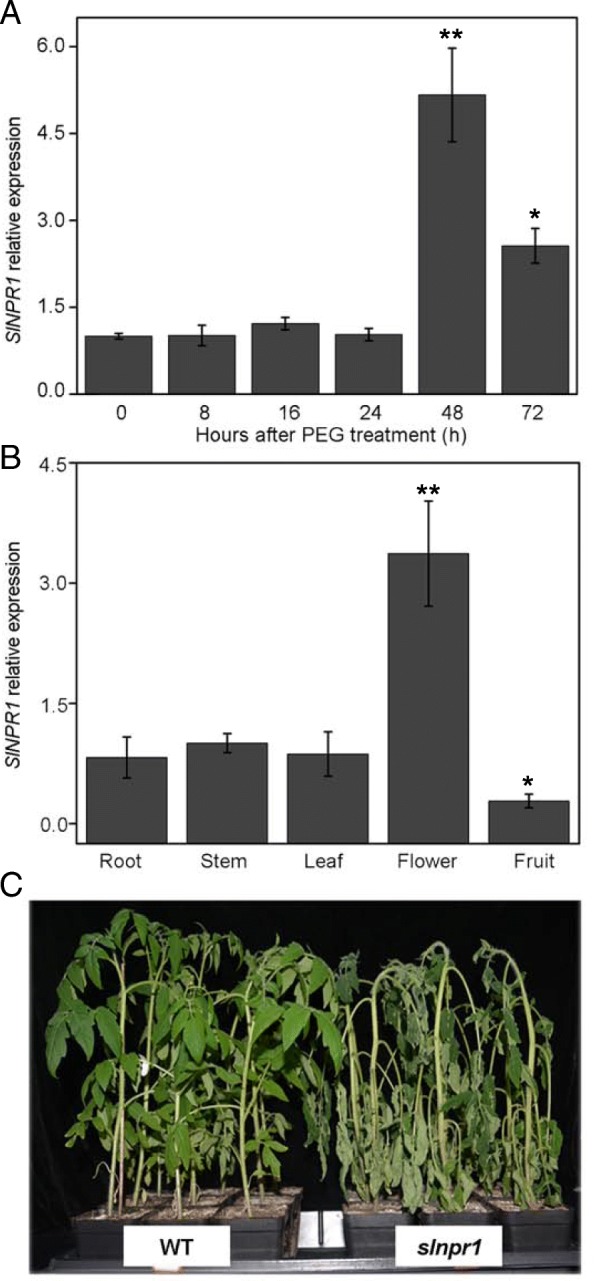
Expression patterns and phenotype under drought stress. (**a**) Expression patterns of *SlNPR1* in WT plants within 3 days after PEG treatment. (**b**) Relative expression of *SlNPR1* in different tissues of WT plants. The error bars indicate the standard deviations of three biological replicates. Asterisks indicate significant differences as determined by Student’s t-test (*, *P* < 0.05; **, *P* < 0.01). (**c**) Phenotype of *slnpr1* mutants and WT plants under drought stress. Photographs were taken 6 days after stopping watering.

Tomato plants under drought stress exhibited a fluctuating *SlNPR1* expression, and the maximum value (5.17-fold) was observed at 48 h after drought stress (Fig. 3a, *P* < 0.01). This result indicates that *SlNPR1* might be involved in response to drought stress. Additionally, transcription level of *SlNPR1* in different tissues was measured to study whether it has any tissue specificity. The samples of root, stem, and leaf were detached from six-week-old WT plants, flower samples were collected when the petals were fully extended, and the fruits samples were collected on 45 days after flowering. Results showed that *SlNPR1* is expressed in all tissues examined, with the highest expression in flowers (Fig. 3b, *P* < 0.01).

### CRISPR/Cas9-mediated *slnpr1* mutants exhibited reduced drought tolerance

**Fig. 4:**
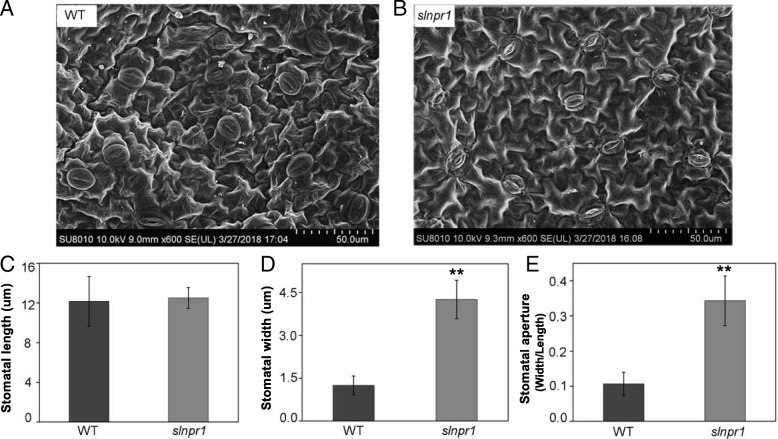
Stomatal aperture of *slnpr1* mutants and wild type (WT) plants under drought stress. Stomatal condition in leaves of (**a**) WT plants and (**b**) *slnpr1* mutants after 3 days’ drought stress. (**c**) Stomatal length, (**d**) stomatal width, and (**e**) stomatal aperture after 3-day drought stress. The error bars indicate the standard deviations of three biological replicates. Asterisks indicate significant differences as determined by Student’s t-test (*, *P* < 0.05; **, *P* < 0.01).

To investigate the role of *SlNPR1* in drought stress further, six-week-old transgenic plants and WT plants were not watered for six consecutive days and photographs were taken at the end of treatment (Fig. 3c). Only a few wilted leaves were found in WT plants. However, *slnpr1* mutants exhibited obvious symptoms: seriously wilted leaves and bent stems. Additionally, the rehydration experiments showed that survival rate of *slnpr1* mutants were significantly lower than that in WT plants (Additional file 5: Figure S3). Furthermore, stomatal aperture in leaves of *slnpr1* mutants and WT plants after 3-day drought stress were investigated using SEM (Fig. 4a and b). The stomatal aperture in *slnpr1* mutants was significantly higher than that in WT plants (Fig. 4e, *P* < 0.05). These results suggest that knockout of *SlNPR1* attenuates tomato plant drought tolerance and negatively regulates stomatal closure under drought stress.

### Characterization of CRISPR/Cas9-mediated mutants based on electrolytic leakage, H_2_O_2_ content and MDA content after drought stress

**Fig. 5:**
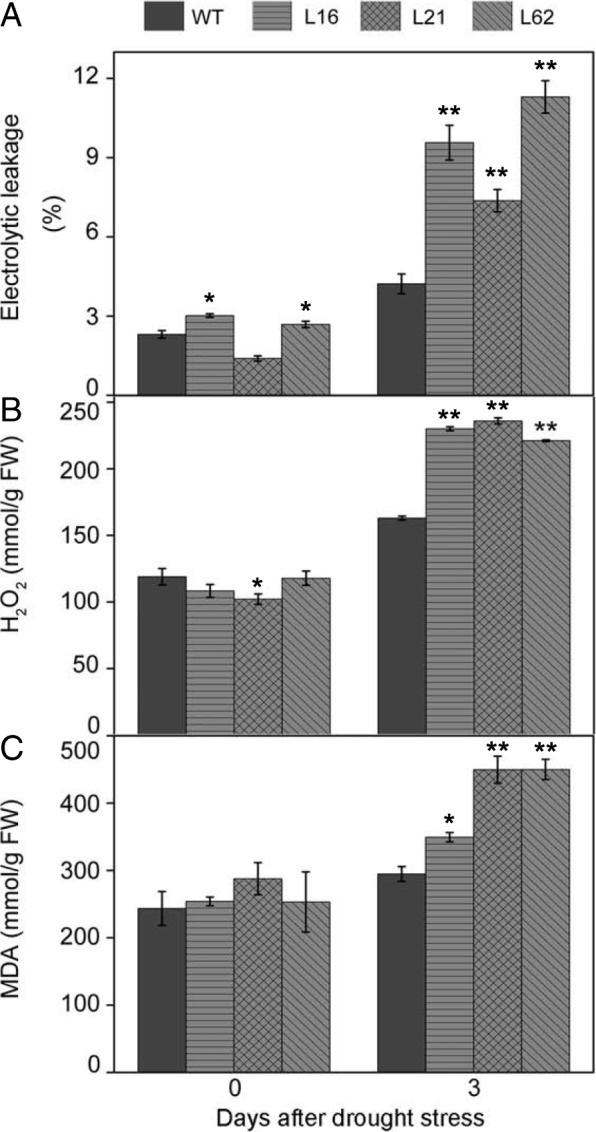
Effects of CRISPR/Cas9-mediated mutations on (**a**) electrolytic leakage, (**b**) hydrogen peroxide (H_2_O_2_), and (**c**) malondialdehyde (MDA) content after drought stress. The error bars indicate the standard deviations of three biological replicates. Asterisks indicate significant differences as determined by Student’s t-test (*, *P* < 0.05; **, *P* < 0.01).

In the present study, electrolytic leakage, H_2_O_2_, and MDA content in both *slnpr1* mutants and WT plants exhibited an increase after 3-day drought stress (Fig. 5). Electrolytic leakage of L16, L21, and L62 was 55%, 42%, and 63% higher than that in WT plants, respectively (Fig. 5a, *P* < 0.01). Meanwhile, higher H_2_O_2_ accumulation was observed in L16, L21, and L62 (230, 236 and 221 mmol·g^−1^ FW, respectively) compared to WT plants (163 mmol·g^−1^ FW) (Fig. 5b, *P* < 0.01). Similarly, *slnpr1* mutants showed a remarkably higher MDA level compared with WT (Fig. 5c, *P* < 0.05).

### Characterization of CRISPR/Cas9-mediated mutants based on APX, SOD, POD, and CAT activities after drought stress

**Fig. 6:**
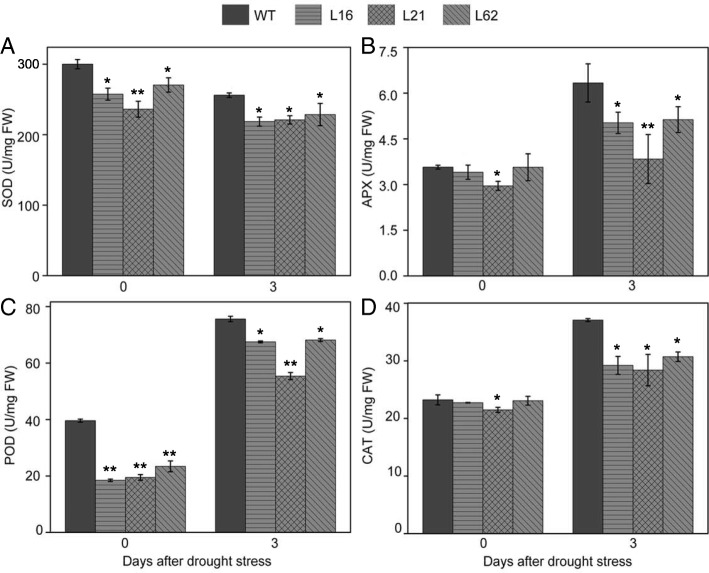
Effects of CRISPR/Cas9-mediated mutations on activities of (**a**) superoxide dismutase (SOD), (**b**) ascorbate peroxidase (APX), (**c**) peroxidase (POD), and (**d**) catalase (CAT) after drought stress. The error bars indicate the standard deviations of three biological replicates. Asterisks indicate significant differences as determined by Student’s t-test (*, *P* < 0.05; **, *P* < 0.01).

The antioxidant enzyme system alleviates the oxidative stress by scavenging ROS, and plays an important role in abiotic stresses, such as drought [36]. Both *slnpr1* mutants and WT plants showed an increase in APX, POD and CAT activities but decrease in SOD activity after 3-day drought stress (Fig. 6). Although SOD activity decreased in both *slnpr1* mutants and WT plants after drought stress, SOD activity in *slnpr1* mutants was still lower than that in WT (Fig. 6a, *P* < 0.05). Knockout of *SlNPR1* significantly decreased APX activity compared to that in WT plants (Fig. 6b, *P* < 0.05). Unlike SOD activity, POD activity clearly increased in both *slnpr1* mutants and WT plants, but it was significantly lower in *slnpr1* mutants than that in WT plants (Fig. 6c, *P* < 0.05). Similarly, on the third day after drought stress, CAT activity in L16, L21, and L62 was 21%, 23% and 17% lower than that in WT plants, respectively (Fig. 6d, *P* < 0.05).

### Characterization of CRISPR/Cas9-meditated mutants on gene expression of *SlGST*, *SlDHN*, and *SlDREB* after drought stress

**Fig. 7:**
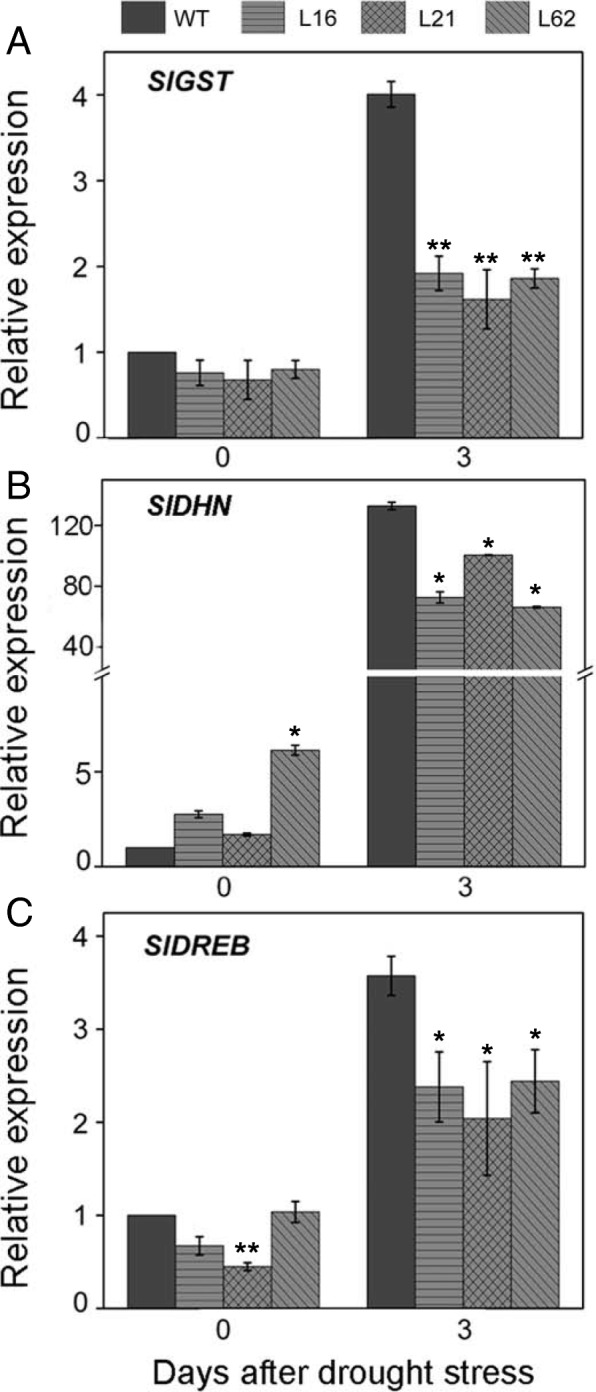
Effects of CRISPR/Cas9-mediated mutants on the relative expression of (**a**) *SlGST* (GenBank ID: XM_004246333), (**b**) *SlDHN* (GenBank ID: NM_001329436), and (**c**) *SlDREB* (GenBank ID: XM_004241698) after drought stress. The *β-Actin* (GenBank ID: NM_001308447) was used as the reference gene. The error bars indicate the standard deviations of three biological replicates. Asterisks indicate significant differences as determined by Student’s t-test (*, *P* < 0.05; **, *P* < 0.01).

To better understand the regulatory mechanism of drought tolerance mediated by *SlNPR1* at molecular level, the expression levels of several drought-related genes were analyzed in both transgenic and WT plants under normal and drought conditions. Comparing with WT plants, the transgenic lines L16, L21, and L62 showed lower expression levels of *SlGST* after 3 days of PEG treatment, and the values were 52%, 60% and 54% lower than that in WT plants, respectively (Fig. 7a, *P* < 0.01). After 3 days’ drought stress, the relative expression of *SlDHN* in *slnpr1* mutants was significantly lower than that in WT (Fig. 7b, *P* < 0.05). Furthermore, knockout of *SlNPR1* significantly decreased relative expressions of *SlDREB* under drought stress, and 3 days after PEG treatment, the expression value in L16, L21, and L62 was 33%, 43% and 32% lower than that in WT, respectively (Fig. 7c, *P* < 0.05).

## Discussion

The function of *AtNPR1* in plant response to biotic stresses has been studied extensively for more than two decades, and the regulatory mechanism has been relatively clear [16–20]. Previous reports have also shown that overexpressing *AtNPR1* in tomato plants enhanced the resistance to a spectrum of fungal and bacterial diseases [37]. However, the research on NPR1’s implication in plant response to abiotic stress is still limited [24]. Recently, *AtNPR1*’s function in plant response to abiotic stress has begun to be concerned [24–28]. Tomato is one of the best-characterized model plants to study gene function [29]. Studying the roles of *SlNPR1* in tomato plant response to abiotic stress not only lays the foundation for cultivating new varieties more suitable for an ever-changing environment, but also aids in expanding understanding of NPR1's mechanism of action.

Phylogenetic analysis showed that two NPR1-like proteins in tomato, SlNML1 and SlNML2, fall within the clade including AtNPR3 and AtNPR4 (Fig. 1a), which are mostly associated with negative SAR regulation [14]. However, SlNPR1 fell within the same clade as AtNPR1, which is mostly recognized as a positive regulator of SAR [13]. This result suggests that the functional characterization of SlNPR1 might be similar to that of AtNPR1 described in previous studies. Moreover, the *cis*-element analysis showed that drought-responsive elements, MYCATRD22 and MYCATERD1, were found within the promoter region of *SlNPR1* (Table 1), suggesting that *SlNPR1* might be involved in response to drought stress. Additionally, relative expression of *SlNPR1* was increased after drought stress (Fig. 3a), which is a second line of evidence suggesting the involvement of *SlNPR1* in modulating plants response to drought stress.

The editing types of T1 generation plants derived from L16, L21, and L62 showed that the edited alleles in T0 generation were inheritable, yet transmission was not completely coincident with Mendelian inheritance. This was supported by previous findings in rice and *A. thaliana* that majority of mutations in early generations occur in somatic cells [38, 39]. In addition, the heterozygous lines of T0 generation carrying wild-type allele were transmitted to T1 generation with some new editing types, and similar result was found in *A. thaliana* [40].

The microstructure of stoma on the leaf surface of *slnpr1* mutants and WT plants was observed, the higher stomatal aperture in *slnpr1* mutants was in agreement with the reports in *A. thaliana* that AtNPR1 played an important role in the stomatal closure signaling pathway [41]. To confirm the remarkably different phenotypes between *slnpr1* mutants and WT plants further (Fig. 3c), physiological and molecular level changes were investigated in the next study. Firstly, cell membranes have been proposed as a primary critical target of environmental stress, and many physiological symptoms caused by such stress are essentially associated with membrane injuries [42]. Electrolytic leakage and MDA content, the indicators of lipid peroxidation and oxidative stress, were measured to evaluate membrane integrity [9, 43]. The higher electrolytic leakage and MDA content in *slnpr1* mutants (Fig. 5a and c) indicated that knockout of *SlNPR1* augmented oxidative damage caused by drought stress. Additionally, membrane damage is always caused by accumulation of ROS under drought stress [44], which is in agreement with the higher H_2_O_2_ content observed in *slnpr1* mutants (Fig. 5b). It suggests that loss of *SlNPR1* function resulted in ROS overproduction, which enhanced the susceptibility to oxidative damage and reduced drought tolerance in tomato plant.

Plants have evolved an efficient antioxidant mechanism to cope with continuous ROS production under environmental stress [45]. The enhanced oxidative stress tolerance in transgenic tobacco plants overexpressing *AtNPR1* was associated with the upregulated genes for APX and Cu^2+^/Zn^2+^SOD [26]. Previous study on tomato plants also reported that induction of antioxidant enzyme activities, including APX, CAT, POD, and SOD, contributed to enhancement of drought tolerance in transgenic plants [46], which indicated that the decreased antioxidant enzymes activities in *slnpr1* mutants (Fig. 6) led to a less efficient ROS scavenging and more severe oxidative damage under drought stress (Fig. 5).

Glutathione-*S*-transferases (GSTs) are a large family of proteins that catalyze the conjugation of GSH to electrophilic substrates and transfer GSH to organic hydro peroxides such as lipid peroxides [47]. Overexpression of *GST* from soybean and *Prosopis juliflora* in tobacco plants resulted in enhanced tolerance to drought stress [48, 49]. Moreover, previous studies in tomato and rice showed that *GST* could positively participate in ROS scavenging [50, 51]. These data support the exhibition of decreased *SlGST* transcript level and higher H_2_O_2_ level in drought-sensitive *slnpr1* mutants (Figs. 5b and 7a). The DREB has been reported to be induced by different abiotic stresses, and it always acted as a positive regulator in drought stress responses [49]. Our results showed that relative expression of *SlDREB* was suppressed notably in *SlNPR1* transgenic lines, which indicated that *SlNPR1* might mediate drought tolerance of tomato plants by regulating the transcription of *SlDREB* (Fig. 7c). Sarkar et al. showed that in peanut *AtDREB* conferred tolerance to drought and salinity stress by reducing the membrane damage and improving ROS scavenging [49], which was in agreement with the increased electrolytic leakage, MDA and H_2_O_2_ contents in our results (Figs. 5 and 7c). Additionally, reports have shown that *SlDREB3* is involved in several ABA-regulated processes through controlling ABA level, and it may encode a factor that is most likely a central component in ABA response machinery [52]. Furthermore, ABA signaling pathway plays an important role in the regulation of the plant's water status during a plant's life cycle [53]. *Dehydrins* (*DHN*) gene is a downstream gene of ABA signaling, which contributes to maintaining stable cell structure in a dehydrated plant [54]. The drought-sensitive *slnpr1* mutants exhibited a decreased *SlDHN* transcript level (Figs. 3c and 7b), which suggested that ABA signaling pathway might be involved in drought tolerance mediated by *SlNPR1*. Additionally, ABA could trigger the occurrence of a complex series of events leading to stomatal closure under drought stress [53]. In the present study, the increased stomatal aperture indicated that ABA signaling pathway in *slnpr1* mutants could be suppressed, which was supported by the previous reports in *A. thaliana* that *AtNPR1* acts downstream of SA, and upstream of ABA, in the stomatal closure signaling pathway [41]. However, how *SlNPR1* knockout affects ABA signaling pathway under drought stress, as well as the complex relationship between SA and ABA signaling pathway in tomato plant response to drought still need studies.

## Conclusion

In conclusion, we found that *SlNPR1* was strongly induced by drought stress and expressed in the root, stem, leaf, flower, and fruit. Furthermore, *slnpr1* mutants enhanced sensitivity to drought stress with higher H_2_O_2_ and MDA contents and electrolytic leakage, suggesting that *SlNPR1* knock out might result in more severe oxidative damage and cell membrane damage. Down-regulated activity levels of antioxidant enzymes (APX, CAT, POD, and SOD) and relative expression of *SlGST* revealed that loss of *SlNPR1* function led to suppression of antioxidant genes and the antioxidant enzyme system under drought conditions. RT-qPCR analysis revealed that transcription of drought-related genes, including *SlGST*, *SlDHN*, and *SlDREB*, were modulated by *SlNPR1* knockout. Further study will focus on the special relationship between *SlNPR1* and ABA signaling pathway under drought stress. This and further studies will provide insights into *SlNPR1*-mediated regulatory mechanism of drought tolerance, and contribute for better understanding the role of *SlNPR1* in response to abiotic stress.

## Methods

### Plant Materials and Stress Conditions

Tomato (*Solanum lycopersicum*) wild type plants ‘Ailsa Craig’ (AC) were planted in plastic pots (7 cm in diameter) containing substrate, vermiculite and black soil (2:1:1, v/v/v) under normal conditions (25 ± 2 °C, 65-70% relative humidity (RH), and photoperiod of 16 h light/8 h dark). AC seeds were kindly provided by Dr. Jim Giovannoni (Boyce Thompson Institute for Plant Research, Ithaca, NY 14853, USA). Six-week-old transgenic lines and WT plants were used for further experiments.

To detect the expression profiles of *SlNPR1* under drought stress, tomato plants (WT) in pots that were filled with composite substrates were irrigated with 25% (w/v) polyethylene glycol (PEG) 6000. Functional leaves were collected at 0, 8, 16, 24, 48, and 72 h, frozen in liquid nitrogen, and stored at −80 °C for further study. Collection of specimens in this study is complied with the international guideline. Three independent biological replicates were measured.

### Phylogenetic analysis

All sequences mentioned in this study were obtained via the NCBI database (Additional file 1: Table S1). Phylogenetic analysis was carried out using MEGA 5.0 by the Neighbor-Joining (NJ) method; a bootstrap test was performed with 1000 replicates. Exon/intron position and domain composition analysis were visualized using IBS software v1.0. Multiple sequence alignments were conducted using ClustalX 2.01 program. To identify *cis*-elements in the *SlNPR1* promoter region, the 1500bp promoter region upstream of the start codon was analyzed with PLACE (https://sogo.dna.affrc.go.jp/cgi-bin/sogo.cgi?lang=en&pj=640&action=page&page=newplace) and PlantCare (http://bioinformatics.psb.ugent.be/webtools/plantcare/html/).

### pYLCRISPR/Cas9-*SlNPR1* Vector Construction

The CRISPR-GE web tool (http://skl.scau.edu.cn/) was used to select two target sequences for *SlNPR1* [55]. The target sequences were introduced into two single guide RNA (sgRNA) expression cassettes using overlapping PCR. The first round PCR was carried out with primers U-F, N1AtU3dT1^−^ (or N1AtU3bT2^−^), N1gRT1^+^ (or N1gRT2^+^) and gR-R. The secondary PCR was performed with corresponding site-specific primer pairs Pps-GGL/Pgs-GG2 (for Target 1) and Pps-GG2/Pgs-GGR (for Target 2), which included *Bsa*I restriction sites. Finally, two sgRNA expression cassettes were ligated into pYLCRISPR/Cas9Pubi-H vector via Golden Gate ligation method [40]. Oligonucleotide primers used for recombinant pYLCRISPR/Cas9 vector construction are listed in Additional file 6: Table S3.

### Plant Transformation

The confirmed pYLCRISPR/Cas9Pubi-H-*SlNPR1* binary vector was transferred into *Agrobacterium tumefaciens* strain EHA105 by electroporation. Transgenic plants were generated through the Agrobacterium-mediated cotyledon transformation method described by Van et al. [56] Transgenic lines were selected based on hygromycin resistance. After *in vitro* regeneration, all hygromycin-positive plants were planted in soil and grown at 25 °C with a 16/8 h light/dark photoperiod.

### Mutation Identification and Off-Target Analysis

The genomic DNA was extracted from fresh frozen leaves (80-100 mg) with a DNA quick Plant System Kit (TIANGEN Biotech Co. Ltd., Beijing, China). Total DNA from T0 and T1 transgenic plants were amplified with the hygromycin resistance-specific primer pair Hyg for and Hyg rev. PCR products were visualized on 1% TAE agarose gel under non-denaturing conditions.

Total DNA of hygromycin-positive plants was used to amplify the desired fragments across Target 1 with primer pair NT1-F and NT1-R (or Target 2 with primer pair NT2-F and NT2-R). The PCR program was as follows: 94 °C for 3 min; 35 cycles of 94 °C for 30 s, 55 °C for 30 s, and 72 °C for 30 s; 72 °C for 7 min. Finally, PCR products were directly sequenced with primer T1/T2 seq based on the Sanger method (Additional file 7: Table S4). Superimposed sequence chromatograms were decoded by DSDecode (http://skl.scau.edu.cn/).

Off-target analysis was carried out using the CRISPR-GE program to predict the potential off-target sites. Then, the top three possible off-target sites for Target 1 and Target 2 were then selected for further analysis (Additional file 4: Table S2). Ten transgenic plants were randomly chosen for off-target analysis. Total DNA from each plant was used as a template to amplify fragments covering the potential off-target sites with the corresponding primer pairs (Additional file 8: Table S5). PCR products were sequenced and then decoded by DSDecode program.

### Drought Stress

Six-week-old plants of T1 transgenic lines, L16, L21, L62, and WT plants were treated with 25% (w/v) PEG 6000 by watering the roots at 25 °C with a photoperiod of 16/8-h light/dark to analyze drought tolerance. Functional leaves from the same positions on each plant were detached before (day 0) and 3 days after PEG treatment, frozen immediately in liquid nitrogen, and stored at −80 °C for further study. Three biological replicates were carried out in this experiment. Additionally, watering was stopped in fifteen six-week-old plants each for transgenic lines and WT plants to observe the phenotype; photographs of plants with representative symptoms were took 6 days later.

### RNA Isolation and RT-qPCR

Total RNA was isolated from frozen leaf tissues with *EasyPure* Plant RNA Kit (Beijing Transgen Biotech Co. Ltd., Beijing, China) according to the manufacturer’s protocol. RNA integrity was assessed by agarose gel electrophoresis (2%) under non-denaturing conditions and quantified by micro-spectrophotometry (NanoDrop™ 2000, Thermo Scientific, Waltham, England).

The *TranScript* One-Step gDNA Removal and cDNA Synthesis SuperMix kit (Beijing Transgen Biotech Co. Ltd., Beijing, China) was used for synthesizing cDNA from a 2 μg aliquot of total RNA. Next, the obtained cDNA was carried out RT-qPCR with *TransStart* Top Green qPCR SuperMix (Beijing Transgen Biotech Co. Ltd., Beijing, China) using a real-time PCR system (CFX96, Bio-Rad, CA, USA) with a final reaction volume of 10 μl. The thermocycling program was as follows: 95 °C for 3 min, followed by 40 cycles of 95 °C for 15 s, and 60 °C for 30 s. Fluorescence changes were monitored in each cycle and *β-Actin* was used as the reference gene for normalization. The relative expression levels were measured using 2^−ΔΔCt^ analysis [57]. Every experiment included three biological repeats, each with three technical replicates. The gene ID, primer sequence, and amplicon length were listed in Additional file 9: Table S6**.**

### Assay of Electrolytic Leakage

Electrolytic leakage was measured according to a previously described method [58] with slight modifications. Briefly, 20 leaf discs of transgenic lines and WT plants were detached by a 1-cm-diameter stainless steel borer, washed thoroughly with distilled water and immersed in vials containing 40 ml deionized water. The solution was shaken at 200 rpm for 2 hours at 25 °C, and solution conductivity (E1) was detected with a conductivity meter (DDS-11A, Leici Instrument Inc., Shanghai, China). Then, the solution was boiled for 15 min, cooled to room temperature (25 ± 2 °C), and solution conductivity (E2) was measured again. Relative electrical conductivity was calculated as (E1/E2) × 100%. This experiment was repeated three times and three biological replicates were carried out.

### MDA and H_2_O_2_ Content

The level of lipid peroxidation was quantified by assessing MDA content using a procedure based on a previous method [59]. Absorbance was recorded at 532 nm and corrected for nonspecific absorbance at 600 nm. Quantity of MDA was calculate using an extinction coefficient of 155 mM^−1^ cm^−1^, and expressed as mmol·g^−1^ fresh weight (FW). H_2_O_2_ content was measured using H_2_O_2_ Detection Kit (A064, Jiancheng, Nanjing, China) according to the operating instructions and was expressed as mmol·g^-1^ FW. Each experiment was repeated three times and three biological replicates were carried out.

### Antioxidant Enzyme Activities

For analysis of ascorbate peroxidase (APX, EC 1.11.1.11), superoxide dismutase (SOD, EC 1.15.1.1), peroxidase (POD, EC 1.11.1.7), and catalase (CAT, EC 1.11.1.6), frozen leaves tissue (0.4 g) in powder was vigorously mixed with 4 ml of cold 100 mM PBS (pH 7.0) using the IKA Disperser [43]. The homogenate was centrifuged at 12, 000 × g for 15 min at 4 °C, and the supernatant was collected for subsequent analysis [60]. APX activity was determined by measuring the oxidation rate of ascorbate at 290 nm [61]. One unit of APX activity was expressed as the quantity of enzyme that oxidized 1 μmol of ascorbate per minute. SOD activity was analyzed using a SOD Detection Kit (A001, Jiancheng, Nanjing, China) by the riboflavin oxidase-nitro blue tetrazolium method, and one unit of SOD activity was defined as the amount of enzyme required to inhibit 50% nitro blue tetrazolium. POD activity was assayed at 470 nm based on a previously described method using guaiacol as a donor and H_2_O_2_ as a substrate [62]. One unit of POD activity was defined as the quantity of enzyme increasing absorbance by 1 per minute. CAT activity was measured by monitoring the rate of H_2_O_2_ decomposition at 240 nm [63]. One unit of CAT activity was defined as the amount of enzyme that decomposed 1 μmol of H_2_O_2_ per minute. Enzyme activity was expressed as U·mg^-1^ FW. Absorbance was recorded using a microplate reader (Infinite M200 Pro, Tecan, Switzerland).

### Scanning Electron Microscopy

After 3 days’ drought stress, the leaves detached from 6-week-old wild-type and transgenic plants were detached and fixed in 2.5% glutaraldehyde. Leaves were then rinsed three times with 0.1 M phosphate buffer (pH 7.2), and serially dehydrated in ethanol (30, 50, 70, 80, 95, 100%). These fixed and dehydrated samples were critical-point dried with CO_2_, sputter-coated with a thin layer of gold and used for stomatal observation using a Hitachi SU8010 scanning electron microscope (Hitachi, Tokyo, Japan). Stomatal length and width were measured from the digital photographs using ImageJ software (https://imagej.nih.gov/ij/download.html). Stomatal aperture was evaluated and calculated by the width/length ratio.

### Statistical Analysis

All data is expressed as mean ± standard deviation (SD). Student’s t-test (*, *P* < 0.05; **, *P* < 0.01) was used for statistical evaluations using SPSS 19.0 (IBM Corporation, Armonk, NY).

## Additional files

Additional file 1: **Table S1.** NPR1 homologous proteins investigated in this study. (DOCX 17 kb)

Additional file 2: **Figure S1.** Multiple sequence alignments of NPR proteins identified in tomato and *Arabidopsis thaliana*. (DOCX 943 kb)

Additional file 3: **Figure S2.** Genome editing type of 26 CR-*NPR1* mutants. (DOCX 1857 kb)

Additional file 4: **Table S2.** Detection of mutations on the putative off-target sites in CR-*SlNPR1* mutants. (DOCX 16 kb)

Additional file 5: **Figure S3.** Survival rate of *slnpr1* mutants and WT plants after re-watering. (DOCX 6731 kb)

Additional file 6: **Table S3.** Oligonucleotide primers used for recombinant pYLCRISPR/Cas9 vector construction. (DOCX 15 kb)

Additional file 7: **Table S4.** Oligonucleotide primers used in mutation detection. (DOCX 15 kb)

Additional file 8: **Table S5.** Oligonucleotide primers used for off-target sites mutation analysis. (DOCX 15 kb)

Additional file 9: **Table S6.** Oligonucleotide primers used for RT-qPCR. (DOCX 15 kb)

## Abbreviations

APX: Ascorbate peroxidase
CAT: Catalase
CRISPR/Cas9: The clustered regularly interspaced short palindromic repeats/CRISPR-associated protein-9 nuclease
DHN: Dehydrin
DREB: Dehydration responsive element binding protein
FW: Fresh weight
GST: Glutathione-*S*-transferases
H_2_O_2_: Hydrogen peroxide
MDA: Malondialdehyde
NPR1: Nonexpressor of pathogenesis-related gene 1
PBS: Phosphate buffered saline
POD: Peroxidase
ROS: Reactive oxygen species
SEM: Scanning electron microscopy
SOD: Peroxide dismutase
