# Supplementary material for: CRISPR/Cas9-Mediated SlNPR1 mutagenesis reduces tomato plant drought tolerance
Source: BMC Plant Biol. 2019 Jan 22;19:38. doi: 10.1186/s12870-018-1627-4 (PMC6341727; doi:10.1186/s12870-018-1627-4)
Supplement: Supplementary file 1 — Table S1. NPR1 homologous proteins investigated in this study. (DOCX 17 kb) [file 12870_2018_1627_MOESM1_ESM.docx]

**Table S1. NPR1 homologous proteins investigated in this study.**

| **Species** | **Identifier** | **GenBank number** |
| --- | --- | --- |
| *Solanum lycopersicum* | SlNPR1 | NP_001234558 |
| *S. lycopersicum* | SlNML1 | NP_001234562 |
| *S. lycopersicum* | SlNML2 | NP_001233844 |
| *Arabidopsis thaliana* | AtNPR1 | NP_176610 |
| *A. thaliana* | AtNPR2 | NP_194342 |
| *A. thaliana* | AtNPR3 | NP_199324 |
| *A. thaliana* | AtNPR4 | NP_193701 |
| *Oryza sativa* | OsNPR1 | XP_015622114 |
| *O. sativa* | OsNPR3 | XP_015620897 |
| *O. sativa* | OsNPR5 | XP_015627734 |
| *O. sativa* | OsNPR6 | XP_015619624 |
| *Nicotiana tabacum* | NtNPR1 | NP_001313196 |
| *N. tabacum* | NtNPR3 | NP_001313050 |
| *N. tabacum* | NtNPR5 | NP_001312808 |
| *Vitis vinifera* | VvNPR1 | XP_002281475 |
| *V. vinifera* | VvNPR2 | XP_010655930 |
| *V. vinifera* | VvNPR3 | XP_002274045 |
| *V. vinifera* | VvNPR5 | XP_002275980 |
| *Brassica napus* | BnNPR1 | XP_013750290 |
| *B. napus* | BnNPR2 | XP_013686075 |
| *B. napus* | BnNPR3 | XP_013687354 |
| *B. napus* | BnNPR4 | XP_013660885 |
| *B. napus* | BnNPR5 | XP_013691750 |
| *B. napus* | BnNPR6 | XP_013746692 |
| *Capsicum annuum* | CaNPR1 | NP_001312028 |
| *C. annuum* | CaNPR3 | XP_016559300 |
| *C. annuum* | CaNPR5 | XP_016543753 |
| *C. annuum* | CaNPR6 | XP_016544908 |
| *Zea mays* | ZmNPR1 | NP_001147587 |
| *Z. mays* | ZmNPR3 | ONM11882 |
| *Z. mays* | ZmNPR5 | AQK38711 |
| *Glycine max* | GmNPR1-1 | NP_001238658 |
| *G. max* | GmNPR1-2 | NP_001238674 |
| *G. max* | GmNPR3 | XP_014622707 |
| *G. max* | GmNPR5 | XP_003518145 |
