# Supplementary material for: CRISPR/Cas9-Mediated SlNPR1 mutagenesis reduces tomato plant drought tolerance
Source: BMC Plant Biol. 2019 Jan 22;19:38. doi: 10.1186/s12870-018-1627-4 (PMC6341727; doi:10.1186/s12870-018-1627-4)
Supplement: Supplementary file 2 — Figure S1. Multiple sequence alignments of NPR proteins identified in tomato and Arabidopsis thaliana. (DOCX 943 kb) [file 12870_2018_1627_MOESM2_ESM.docx]

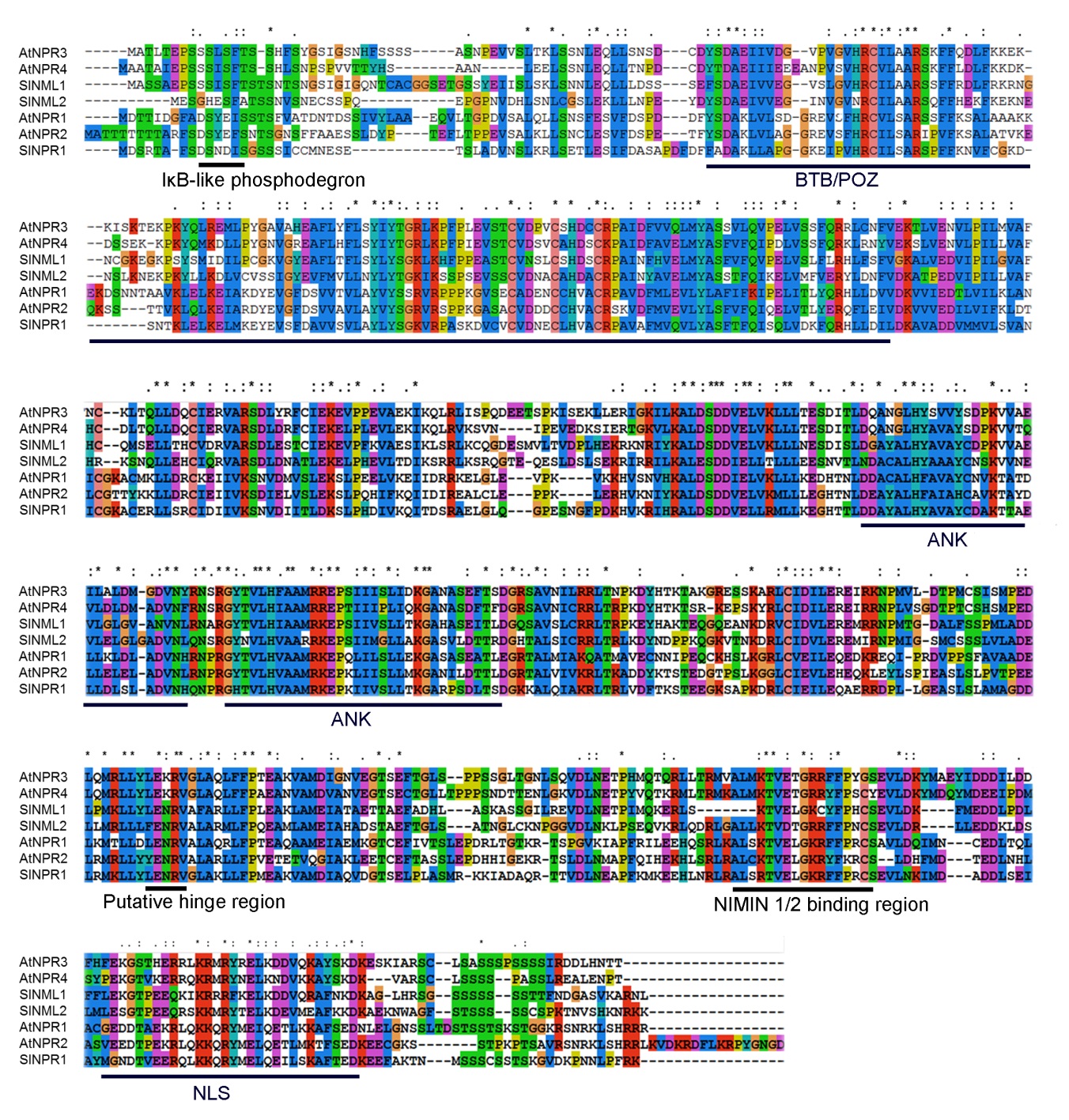


**Fig. S1.** Multiple sequence alignments of NPR proteins identified from tomato and *Arabidopsis thaliana*. Sequences were aligned using ClustalX 2.01 program. Consensus keys: ‘*’, single, fully conserved residue; ‘:’, conservation of strong groups; ‘.’, conservation of weak groups; ‘-’, no consensus.
