# Supplementary material for: CRISPR/Cas9-Mediated SlNPR1 mutagenesis reduces tomato plant drought tolerance
Source: BMC Plant Biol. 2019 Jan 22;19:38. doi: 10.1186/s12870-018-1627-4 (PMC6341727; doi:10.1186/s12870-018-1627-4)
Supplement: Supplementary file 3 — Figure S2. Genome editing type of 26 CR-NPR1 mutants. (DOCX 1857 kb) [file 12870_2018_1627_MOESM3_ESM.docx]

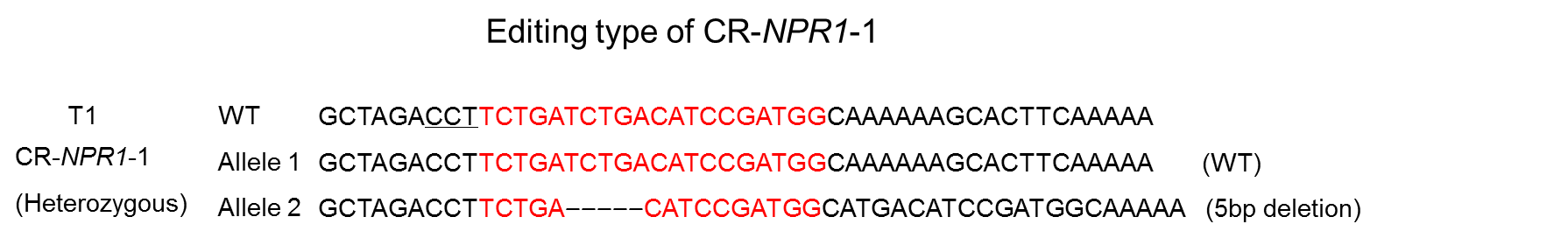


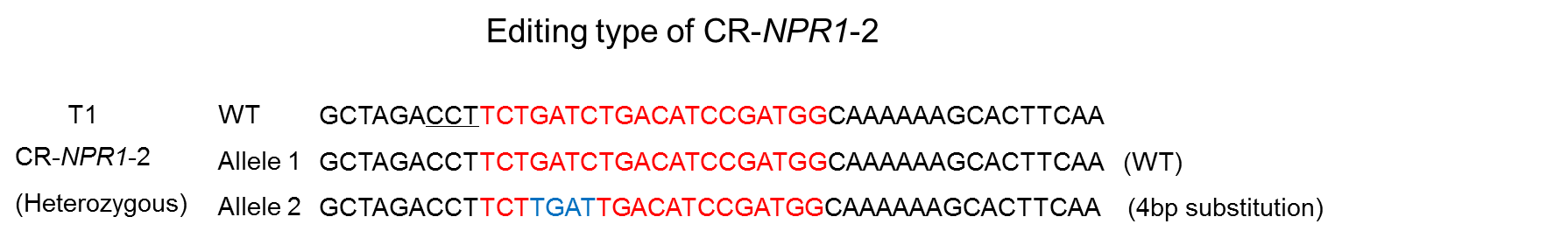


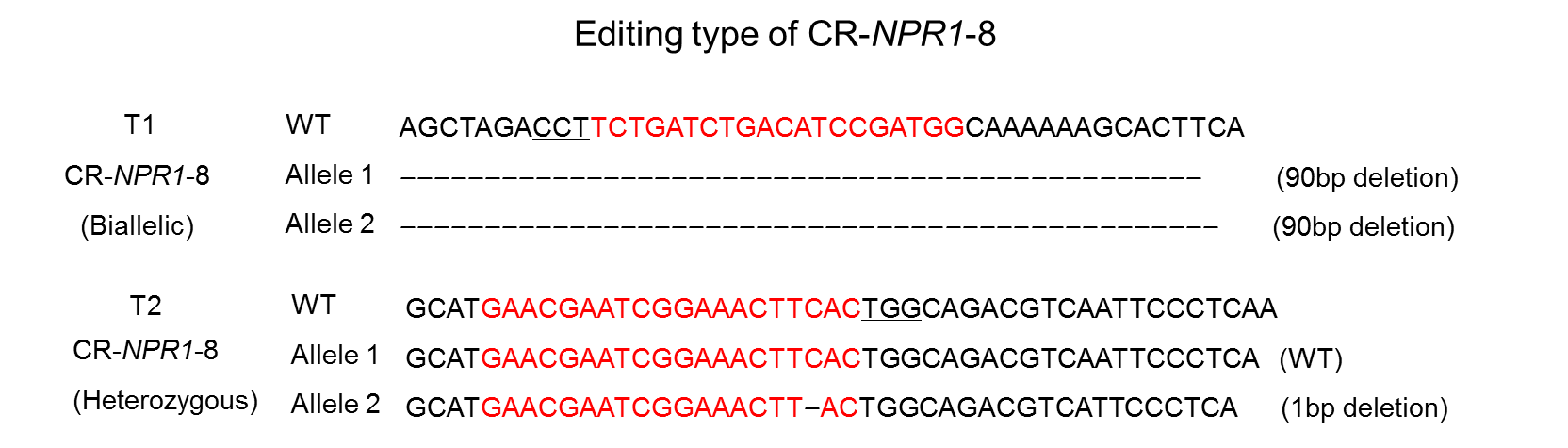


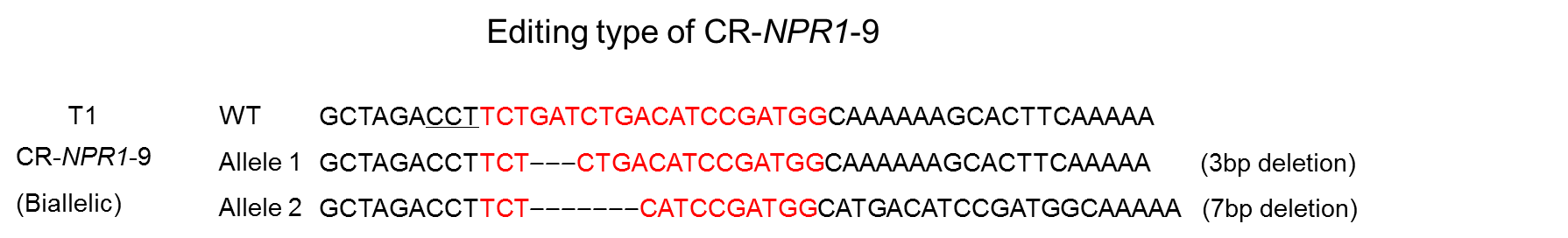


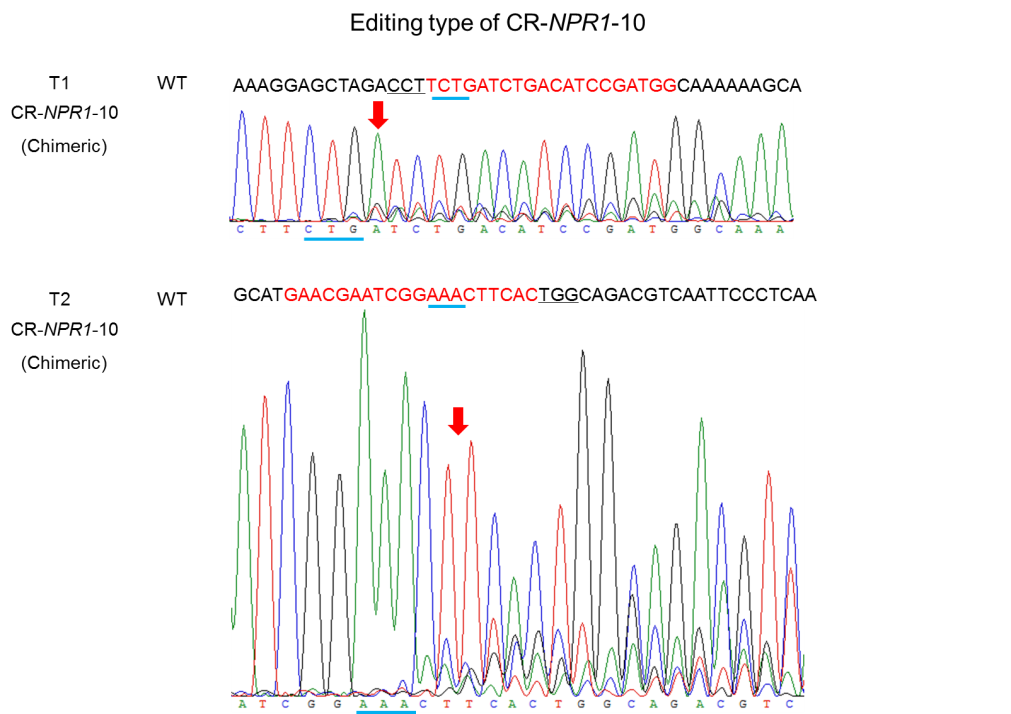


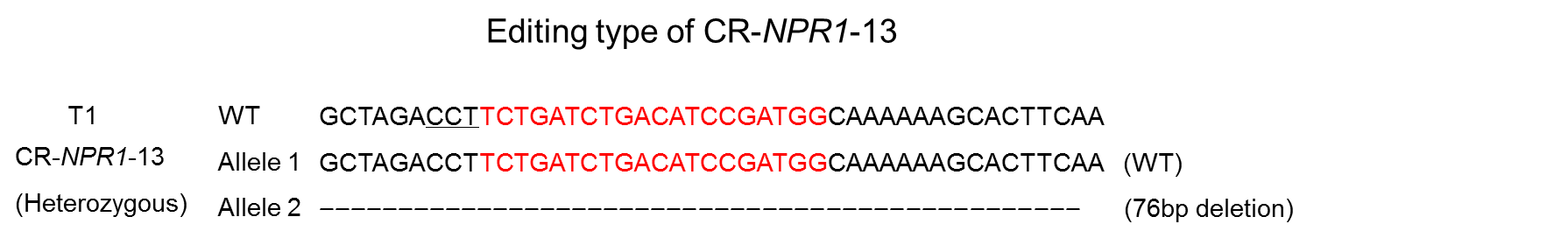


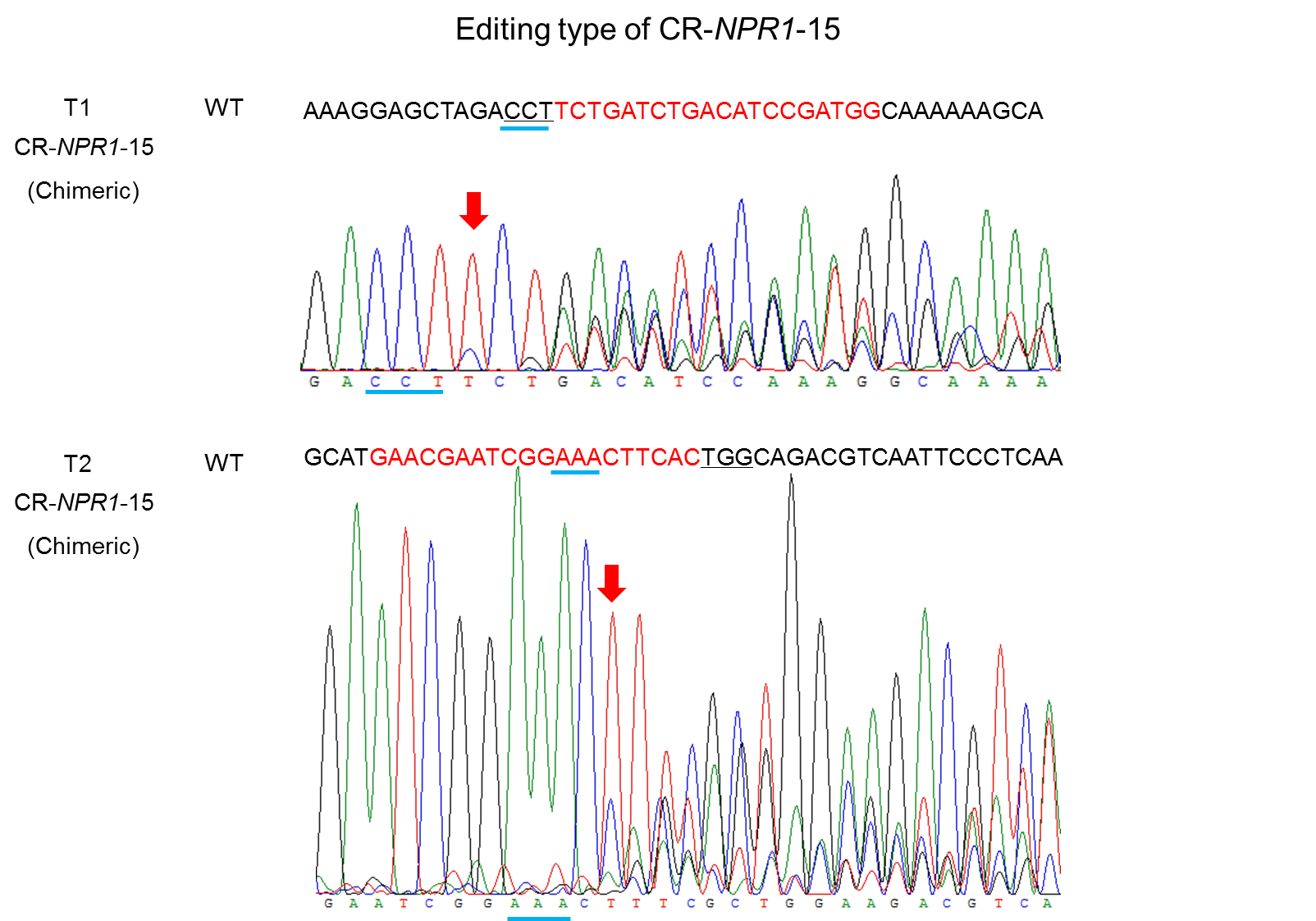


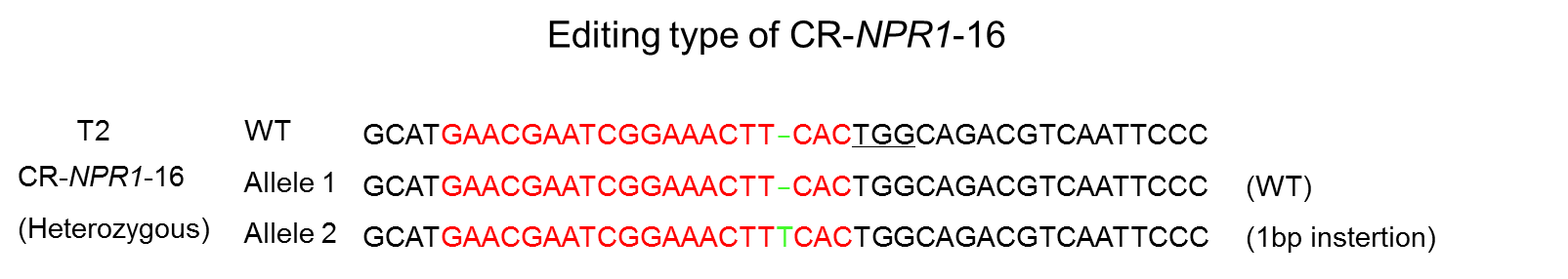


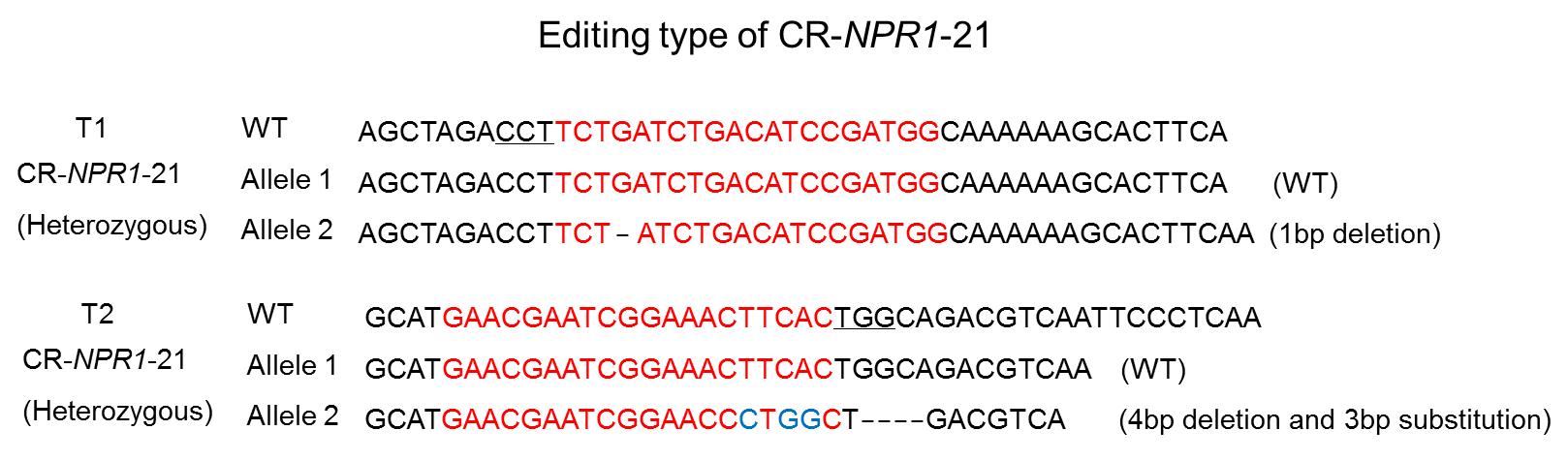


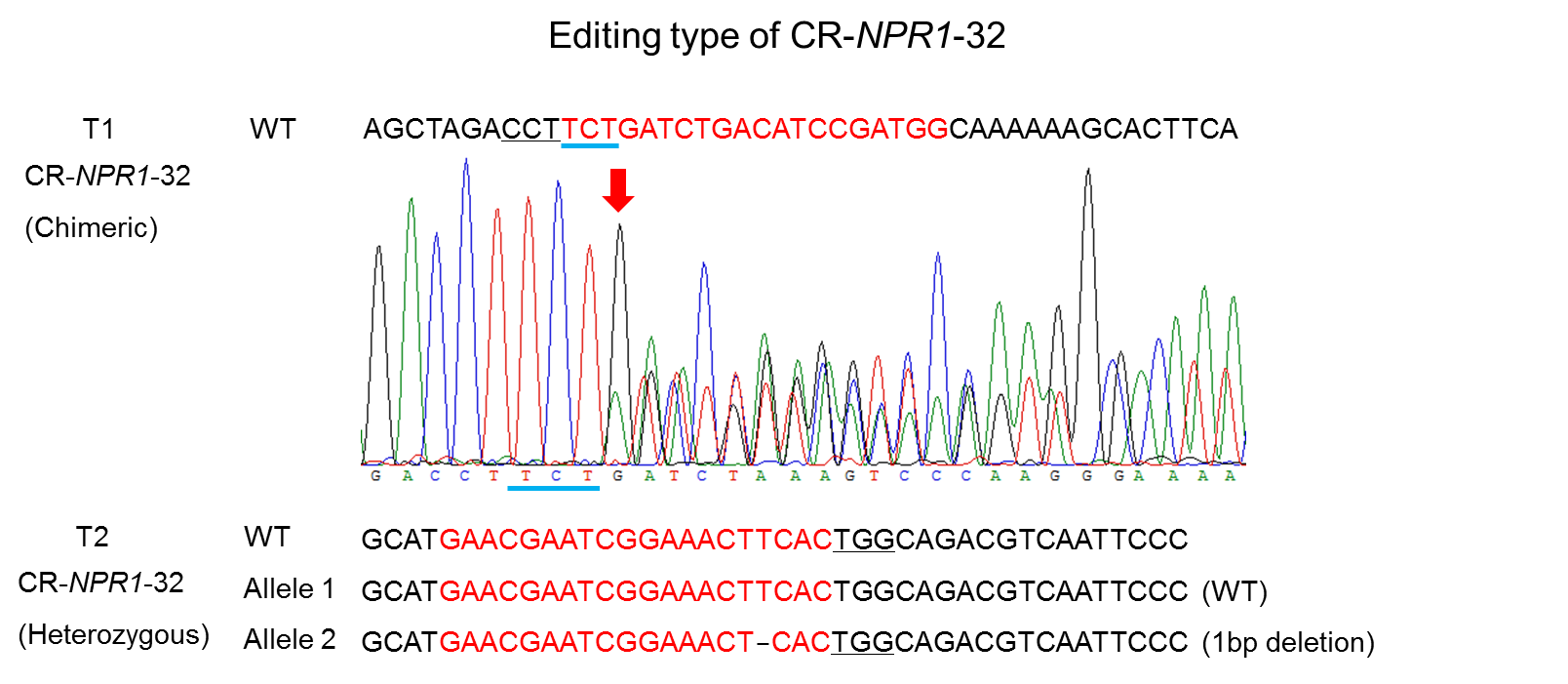


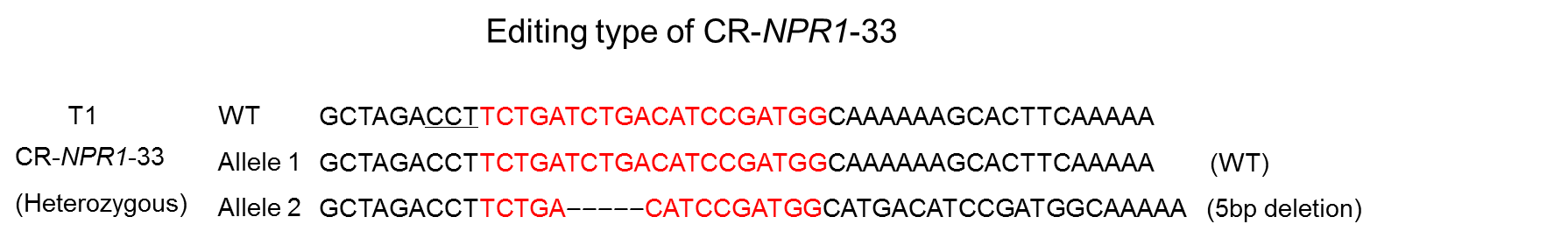


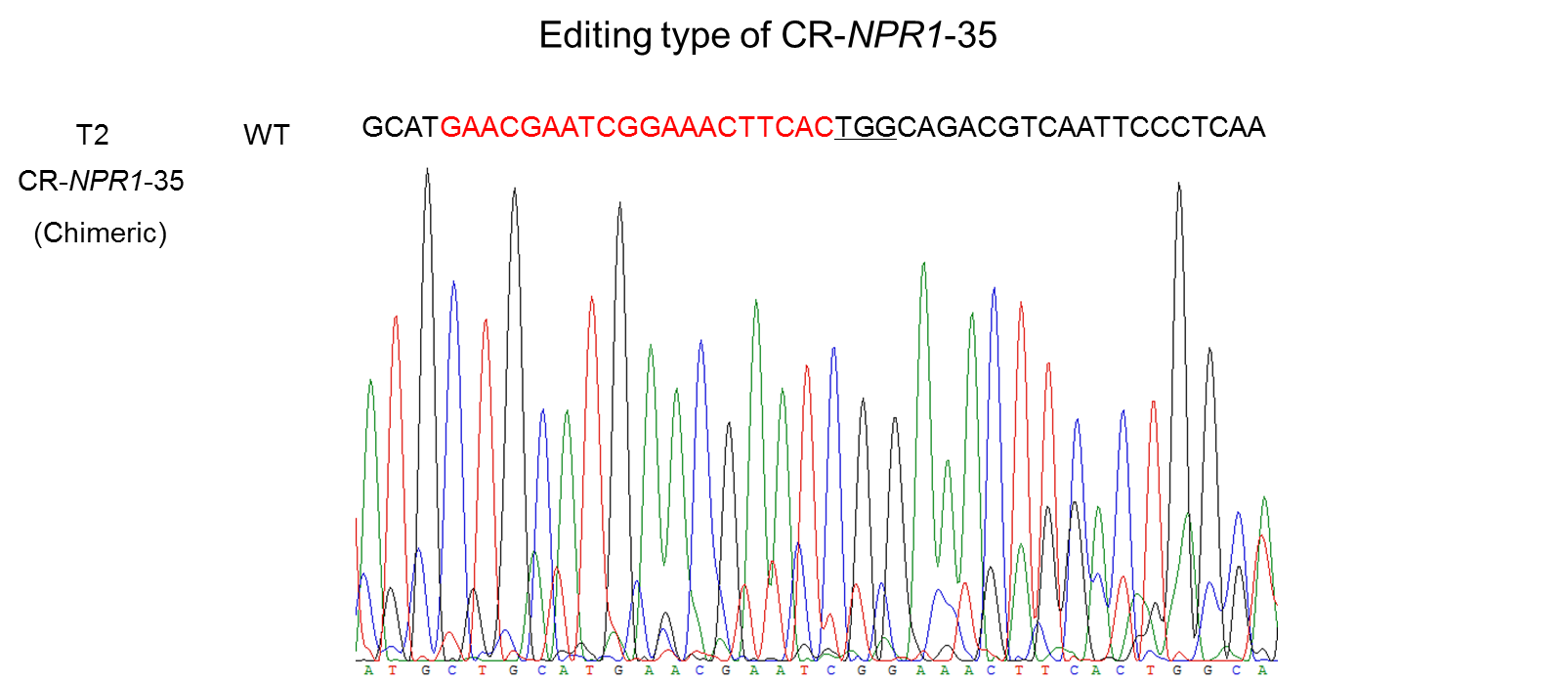


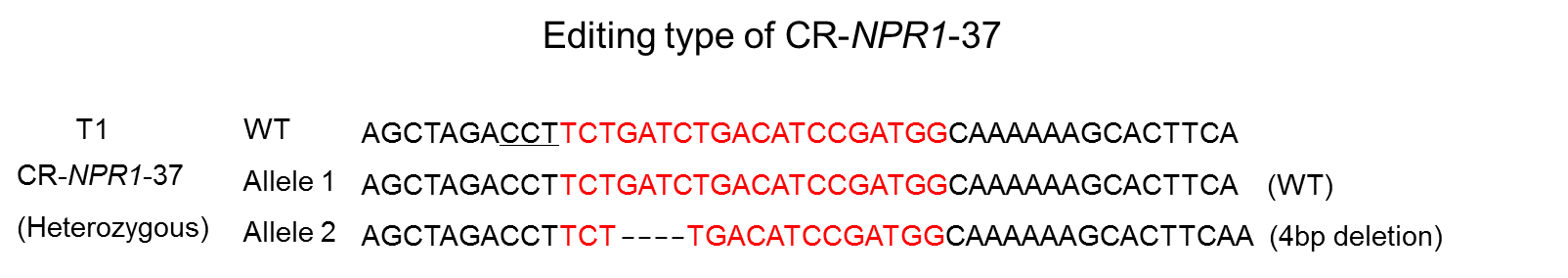


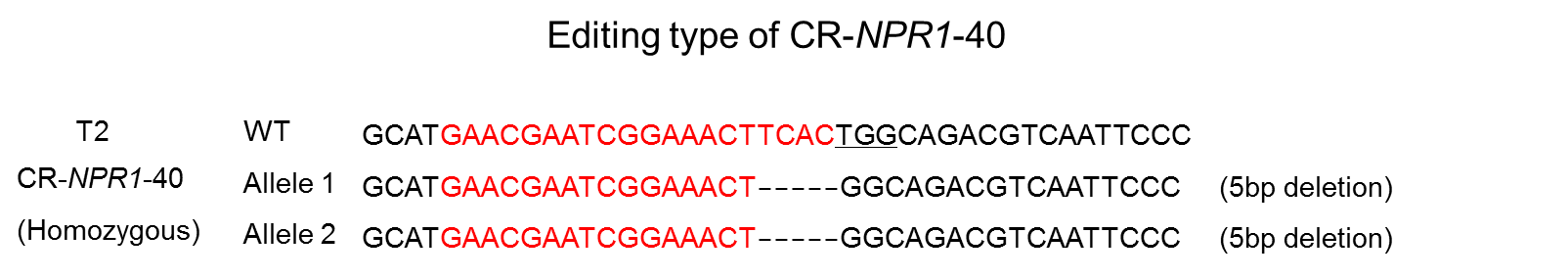


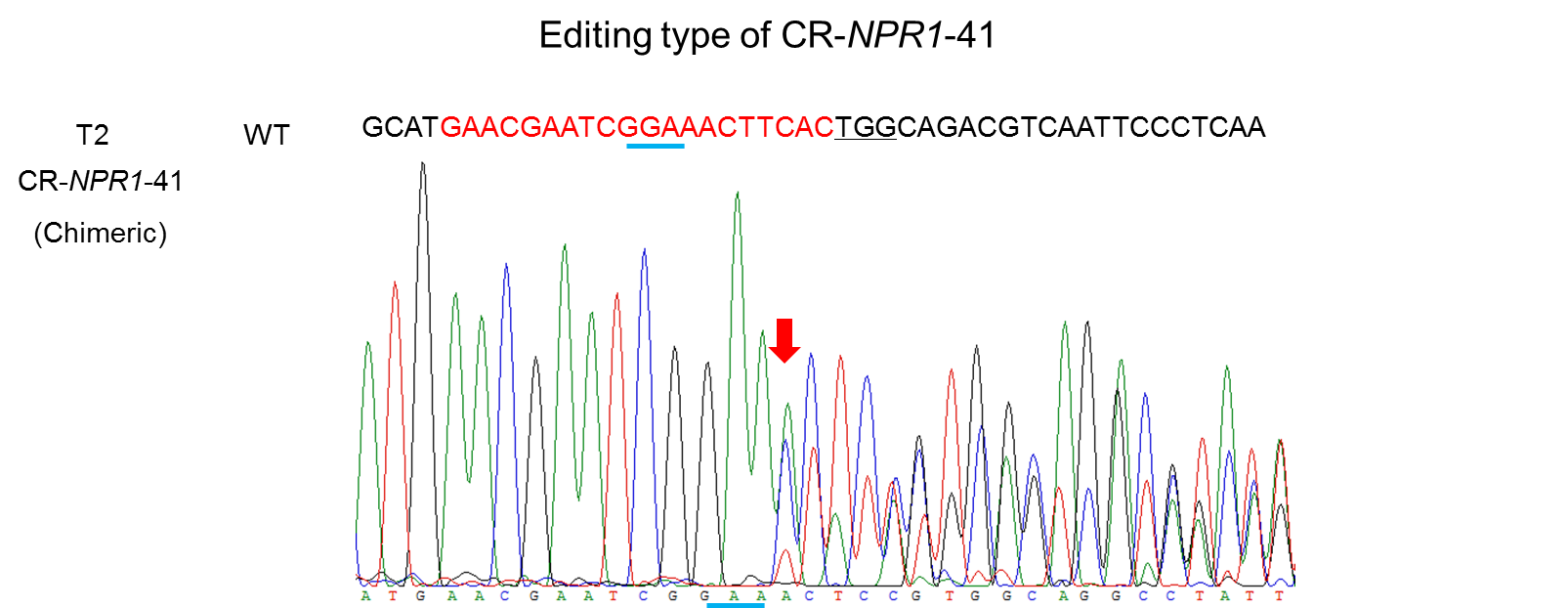


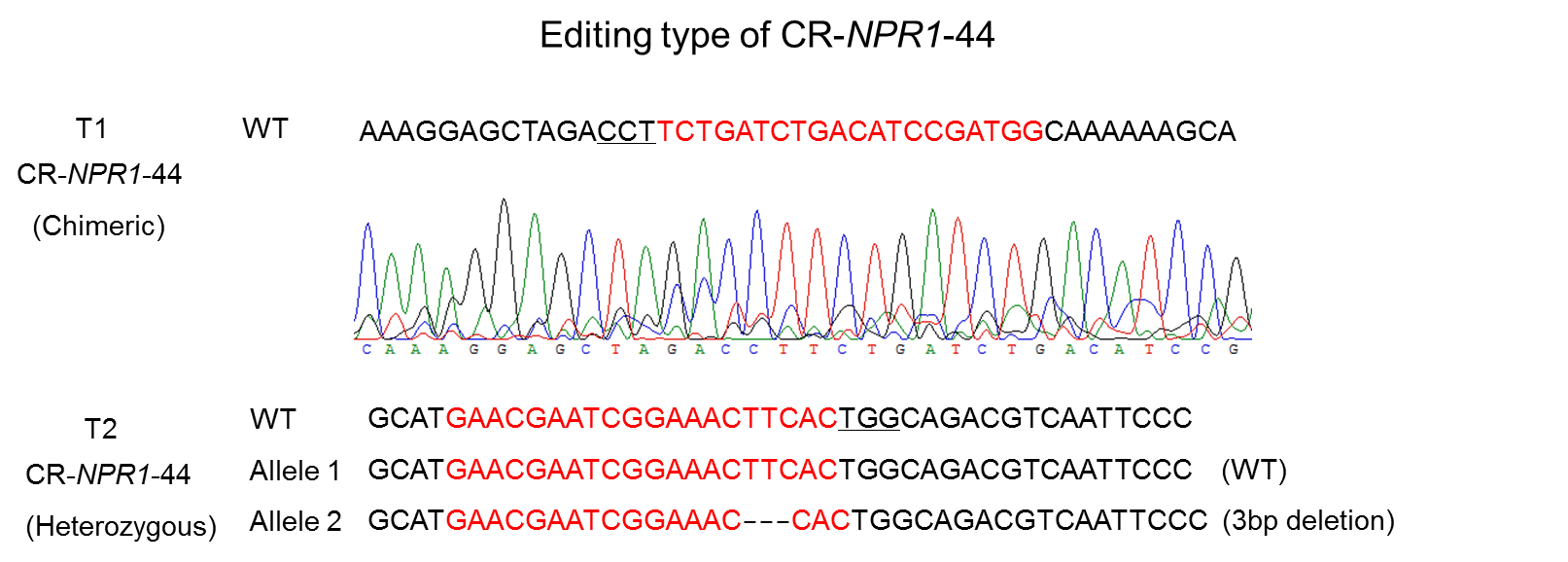


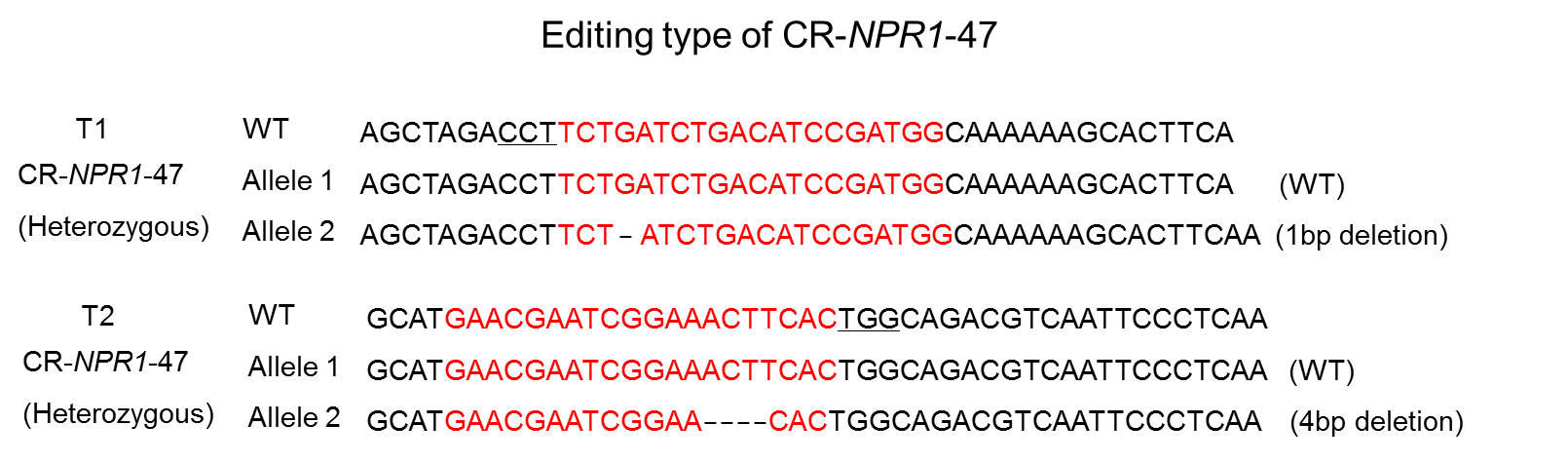


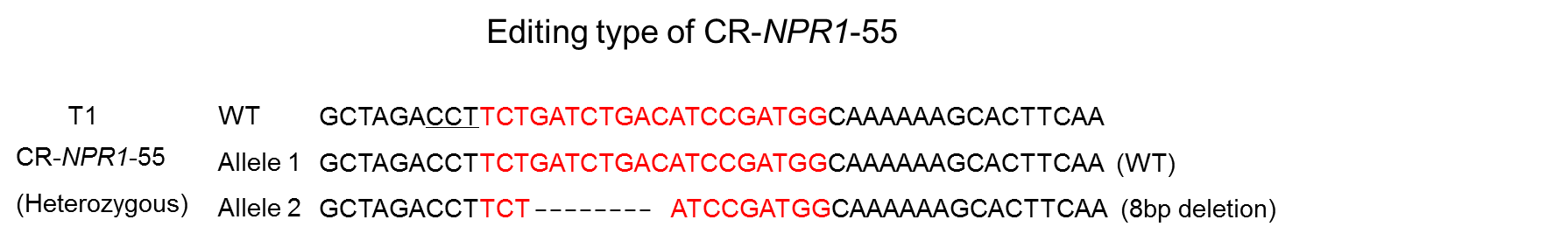


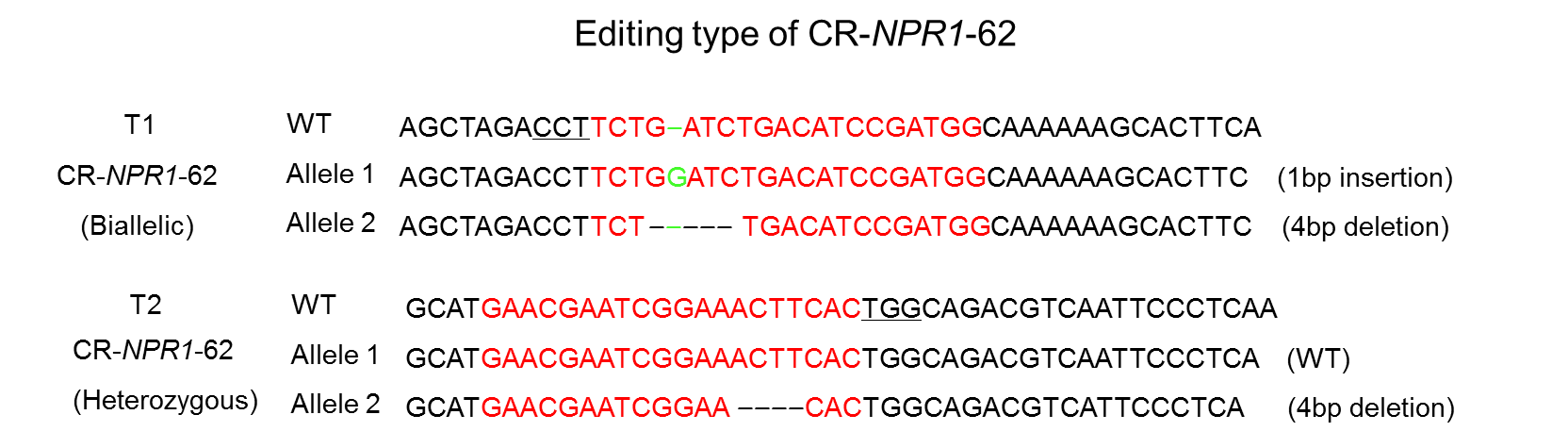


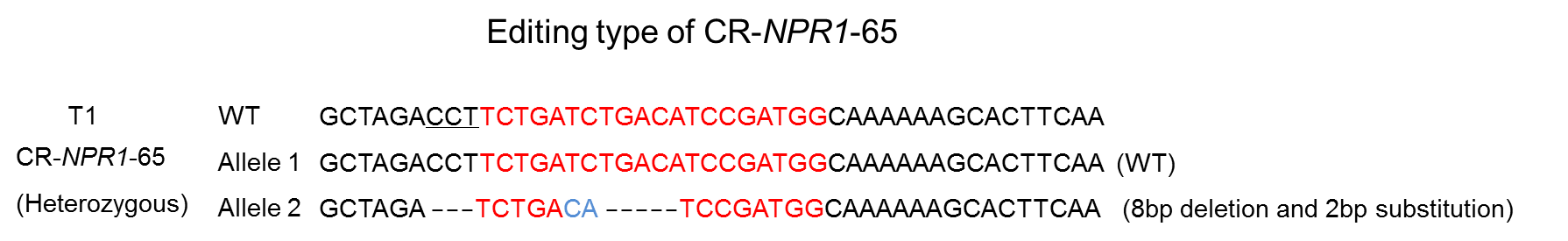


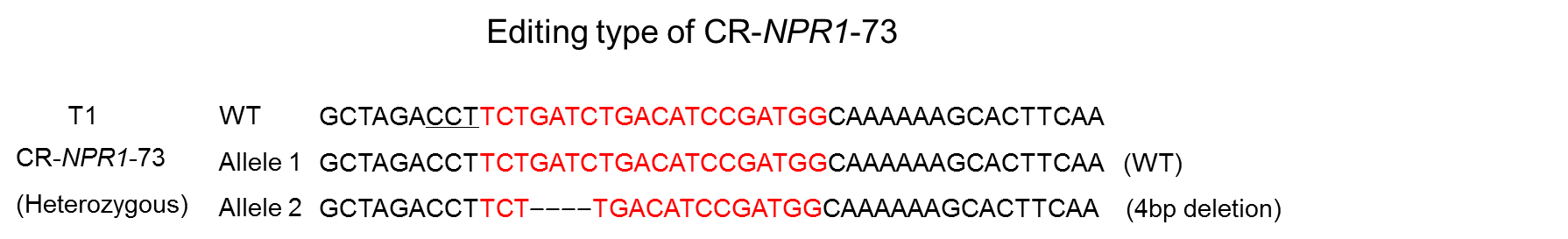


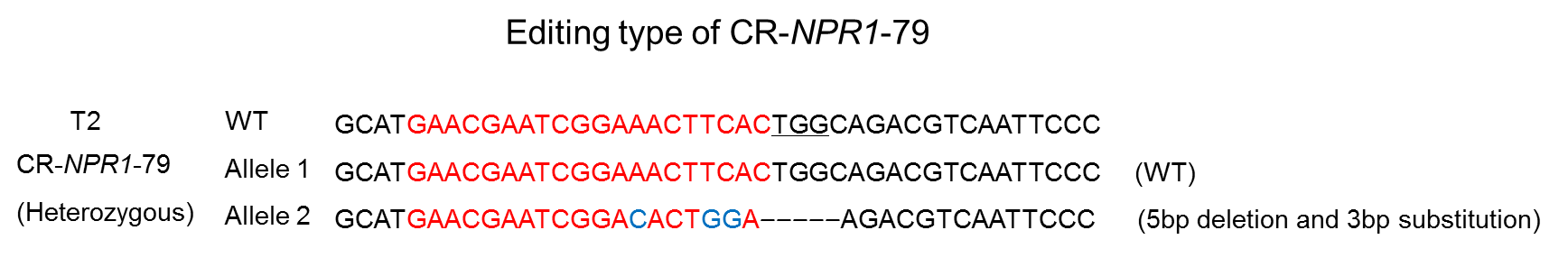


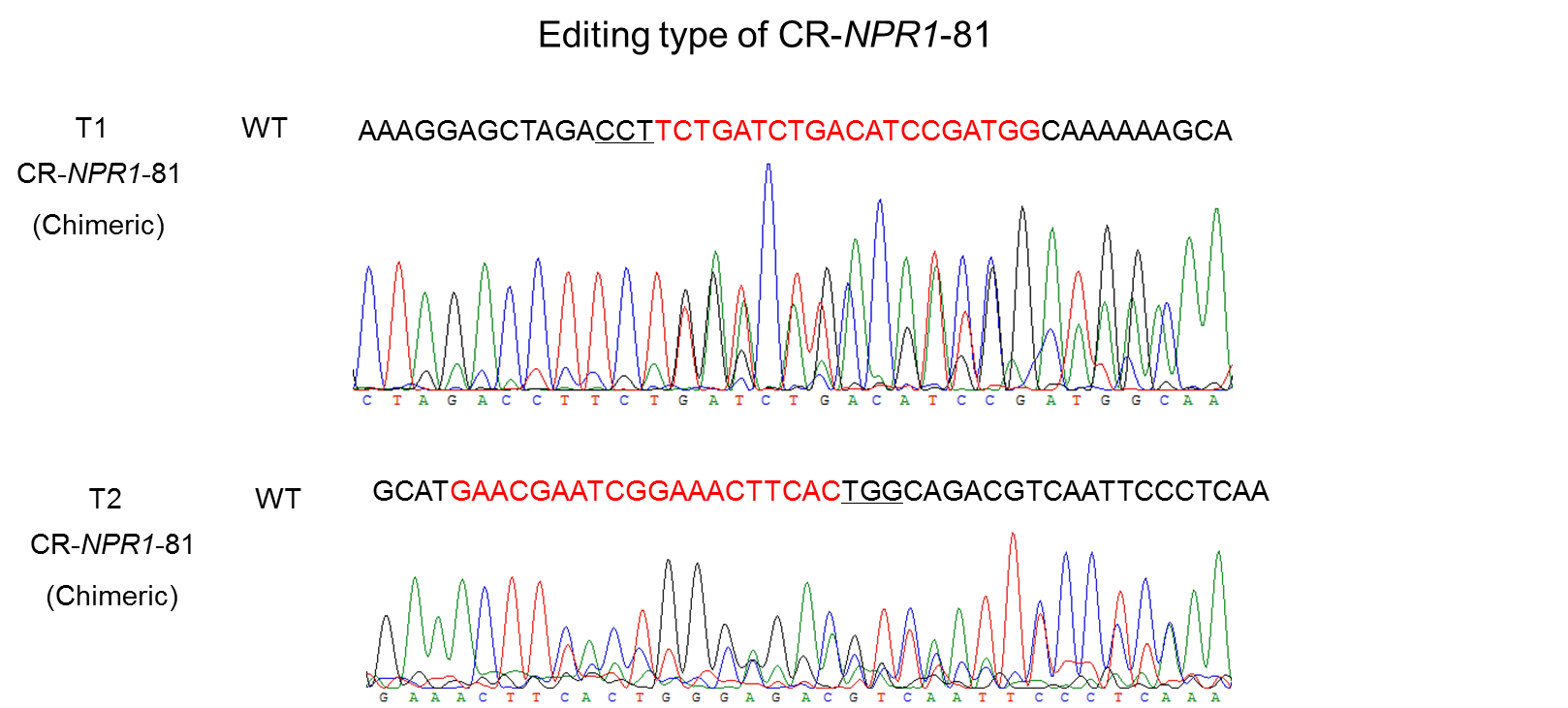


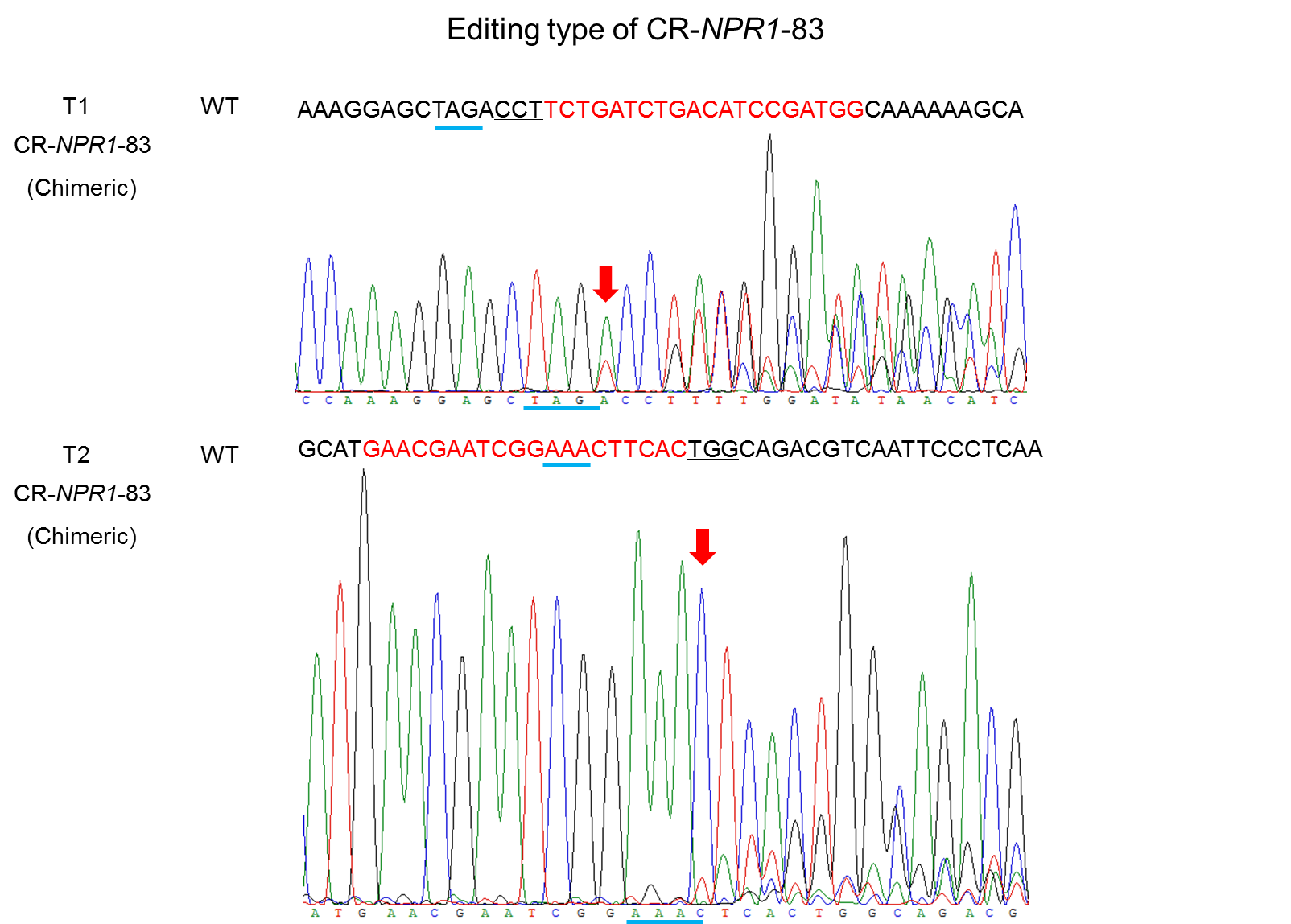


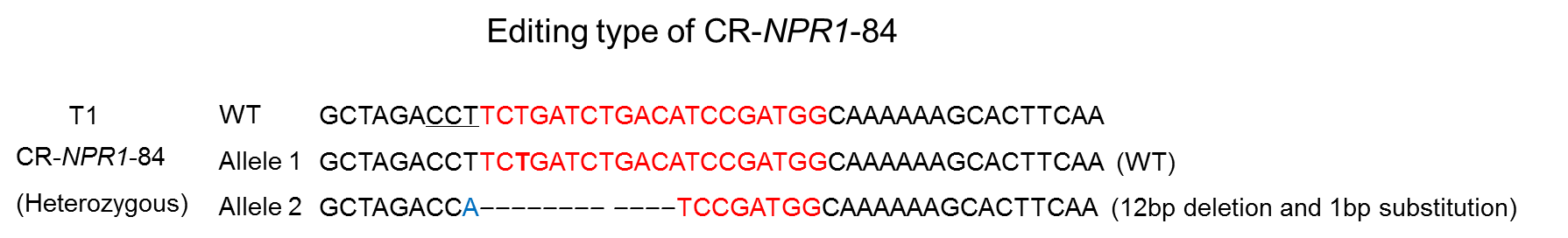


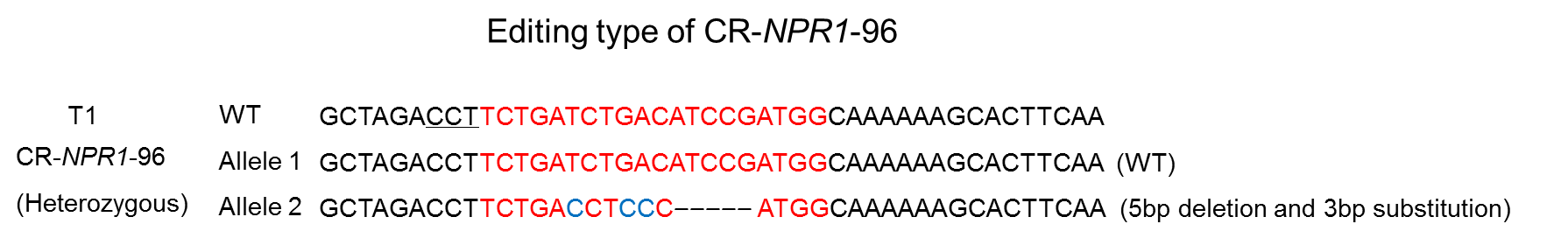


**Fig. S2. Genome editing types of *SlNPR1* mutants.**

Red letters indicate the target sequences, green letters represent base insertion, blue letters represent base substitutions, minus symbols represent deletions. Chimeric mutations were shown with sequencing chromatogram, the letters with blue underline were with single sequencing peak, and the red arrow indicated the beginning of chimeric mutation with multi sequencing peak.
