# Supplementary material for: CRISPR/Cas9-Mediated SlNPR1 mutagenesis reduces tomato plant drought tolerance
Source: BMC Plant Biol. 2019 Jan 22;19:38. doi: 10.1186/s12870-018-1627-4 (PMC6341727; doi:10.1186/s12870-018-1627-4)
Supplement: Supplementary file 4 — Table S2. Detection of mutations on the putative off-target sites in CR-SlNPR1 mutants. (DOCX 16 kb) [file 12870_2018_1627_MOESM4_ESM.docx]

| Target | Name of putative off-target sites | putative off-target locus | Sequence of the putative oﬀ-target site | No. of mismatching bases | *No.of plants sequenced | No.of plants with mutations |
| --- | --- | --- | --- | --- | --- | --- |
|  |  |  |  |  |  |  |
|  |  |  |  |  |  |  |
| Target 1 | OFF1 | ch01:28194567-28194589 | TCATCAAATGTCACATCAGAAGG | 4 | 10 | 0 |
|  | OFF2 | ch10:38812637-38812659 | CCATCAAATGTCAGATAAAATGG | 4 | 10 | 0 |
|  | OFF3 | ch10:46946972-46946950 | CCATATGATGTTAGACCAGAGGG | 4 | 10 | 0 |
| Target 2 | OFF1 | ch04:25992641-25992663 | GATCGACTAGGAAACTTCAATGG | 4 | 10 | 0 |
|  | OFF2 | ch03:51485025-51485003 | TAACCAATTTGAAACTTCACTAG | 4 | 10 | 0 |
|  | OFF3 | ch10:19834545-19834523 | GAACAAATCAGAAACTTAATTGG | 4 | 10 | 0 |

**Table S2. Detection of mutations on the putative oﬀ-target sites in CR-*SlNPR1* mutants.**
