# Supplementary material for: CRISPR/Cas9-Mediated SlNPR1 mutagenesis reduces tomato plant drought tolerance
Source: BMC Plant Biol. 2019 Jan 22;19:38. doi: 10.1186/s12870-018-1627-4 (PMC6341727; doi:10.1186/s12870-018-1627-4)
Supplement: Supplementary file 5 — Figure S3. Survival rate of slnpr1 mutants and WT plants after re-watering. (DOCX 6731 kb) [file 12870_2018_1627_MOESM5_ESM.docx]

**Fig. S3. Survival rate of *slnpr1* mutants and WT plants after re-watering.**

Four-week-old transgenic plants and WT plants were used in the rehydration experiments to calculate the survival rate. Before stopping watering, all plants were grown under normal conditions (25 ± 2 °C, 65-70% relative humidity (RH), and photoperiod of 16 h light/8 h dark), and all groups were irrigated with equal volume of water. “das”, days after stopping watering; “dar”, days after re-watering.
