# Supplementary material for: CRISPR/Cas9-Mediated SlNPR1 mutagenesis reduces tomato plant drought tolerance
Source: BMC Plant Biol. 2019 Jan 22;19:38. doi: 10.1186/s12870-018-1627-4 (PMC6341727; doi:10.1186/s12870-018-1627-4)
Supplement: Supplementary file 6 — Table S3. Oligonucleotide primers used for recombinant pYLCRISPR/Cas9 vector construction. (DOCX 15 kb) [file 12870_2018_1627_MOESM6_ESM.docx]

| **Table S3. Oligonucleotide primers used for recombinant pYLCRISPR/Cas9 vector construction.** | | |
| --- | --- | --- |
| **Purpose** | **Primers** | **Sequence (5'→3')** |
| 1^st^ PCR | U-F | CTCCGTTTTACCTGTGGAATCG |
|  | gR-R | CGGAGGAAAATTCCATCCAC |
|  | N1gRT1^+^ | CCATCGGATGTCAGATCAGAGTTTTAGAGCTAGAAAT |
|  | N1AtU3dT1^-^ | TCTGATCTGACATCCGATGGTGACCAATGGTGCTTTG |
|  | N1gRT2^+^ | GAACGAATCGGAAACTTCACGTTTTAGAGCTAGAAAT |
|  | N1AtU3bT2^-^ | GTGAAGTTTCCGATTCGTTCTGACCAATGTTGCTCC |
| 2^nd^ PCR | Pps-GGL | TTCAGAGGTCTCTCTCGACTAGTATGGAATCGGCAGCAAAGG |
|  | Pgs-GG2 | AGCGTGGGTCTCGTCAGGGTCCATCCACTCCAAGCTC |
|  | Pps-GG2 | TTCAGAGGTCTCTCTGACACTGGAATCGGCAGCAAAGG |
|  | Pgs-GGR | AGCGTGGGTCTCGACCGACGCGTATCCATCCACTCCAAGCTC |

The restriction enzyme *Bsa*I site was underlined.
