# Supplementary material for: CRISPR/Cas9-Mediated SlNPR1 mutagenesis reduces tomato plant drought tolerance
Source: BMC Plant Biol. 2019 Jan 22;19:38. doi: 10.1186/s12870-018-1627-4 (PMC6341727; doi:10.1186/s12870-018-1627-4)
Supplement: Supplementary file 7 — Table S4. Oligonucleotide primers used in mutation detection. (DOCX 15 kb) [file 12870_2018_1627_MOESM7_ESM.docx]

| **Table S4. Oligonucleotide primers used in mutation detection.** | | |
| --- | --- | --- |
| **Purpose** | **Primer** | **Sequence (5'→3')** |
| Transgenic plant identifition | Hyg for | CTTGACATTGGGGAGTTTAGCGAGA |
|  | Hyg rev | CCCTTATCTGGGAACTACTCACACA |
| Mutation detection | NT1-F | CAGATGATGTAATGATGGTT |
|  | NT1-R | ATAGGCTGTCAGAAGAGGAG |
|  | T1 seq | TATGCTCTCCACTATGCTGT |
|  | NT2-F | CTCCATTTCCATTGCTTCCT |
|  | NT2-R | TTCGTGTTGCTATCTTTCCC |
|  | T2 seq | CCCTCTATTCCATTCCCCCT |
