# Supplementary material for: CRISPR/Cas9-Mediated SlNPR1 mutagenesis reduces tomato plant drought tolerance
Source: BMC Plant Biol. 2019 Jan 22;19:38. doi: 10.1186/s12870-018-1627-4 (PMC6341727; doi:10.1186/s12870-018-1627-4)
Supplement: Supplementary file 8 — Table S5. Oligonucleotide primers used for off-target sites mutation analysis. (DOCX 15 kb) [file 12870_2018_1627_MOESM8_ESM.docx]

**Table S5. Oligonucleotide primers used for off-target site mutation analysis.**

| Off-target site | Primers | Sequence (5'→3') |
| --- | --- | --- |
| Target 1- OFF1 | For | GGGGTTACTCTTCTCACACT |
|  | Rev | TCTACATCTACGCCCATATA |
|  | Seq | GGTGCTATCTGAATGCTGGAA |
| Target 1- OFF2 | For | TGTTTTCTCACTCTTCTTTC |
|  | Rev | ACTTGTTTTGTTTATCGTAT |
|  | Seq | TCATTGTAGTCCAGGAGTTC |
| Target 1- OFF3 | For | AAGCACTCCTTACAAAGAAT |
|  | Rev | TGAGGGTAGAAAAAGACACT |
|  | Seq | TCTCATTATCCCATCCACGTT |
| Target 2- OFF1 | For | TAAAATAGCGGTCAAATGTA |
|  | Rev | GAGCAGCAACTGAAAGAGAA |
|  | Seq | ACACCATCAACTCTCTTCCTA |
| Target 2- OFF2 | For | CTTGTATTTTCCTCCTCTTT |
|  | Rev | TATTCTATAACTTCATGGTT |
|  | Seq | TTACGGAGGCTACATAATGG |
| Target 2- OFF3 | For | AGTTGTCTGACTTGTCCTTC |
|  | Rev | TGTTAGTTTTGAGATTTGGT |
|  | Seq | AACGAGTTGATAGCAAGTGT |
