# Supplementary material for: CRISPR/Cas9-Mediated SlNPR1 mutagenesis reduces tomato plant drought tolerance
Source: BMC Plant Biol. 2019 Jan 22;19:38. doi: 10.1186/s12870-018-1627-4 (PMC6341727; doi:10.1186/s12870-018-1627-4)
Supplement: Supplementary file 9 — Table S6. Oligonucleotide primers used for RT-qPCR. (DOCX 15 kb) [file 12870_2018_1627_MOESM9_ESM.docx]

**Table S6. Oligonucleotide primers used for RT-qPCR.**

| **Gene** | **Gene ID** | **Forward primer (5'→3')** | **Reverse primer (5'→3')** | **Amplicon length (bp)** |
| --- | --- | --- | --- | --- |
| *SlGST* | XM_004246333 | TTCCATTTTGCCTAAAGACC | TTCCACACTGCTGACCCCT | 87 |
| *SlDHN* | NM_001329436 | TGGTTTGTTTGATTTCATTG | AGTTTCTTTTCCTCCTCCTT | 138 |
| *SlDREB* | XM_004241698 | GACTCATTGCCTCGCCCA | TTTCACCCAGTTCCTCCG | 154 |
| *β-actin* | NM_001308447 | CAGCAGATGTGGATCTCAAA | CTGTGGACAATGGAAGGAC | 59 |
